# Supplementary material for: A high-quality human reference panel reveals the complexity and distribution of genomic structural variants
Source: Nat Commun. 2016 Oct 6;7:12989. doi: 10.1038/ncomms12989 (PMC5059695; doi:10.1038/ncomms12989)
Supplement: Supplementary Information — Supplementary Figures 1-11, Supplementary Tables 1-5, Supplementary Methods and Supplementary References. [file ncomms12989-s1.pdf]

1 Supplementary Figures

1.1 Supplementary Figure 1

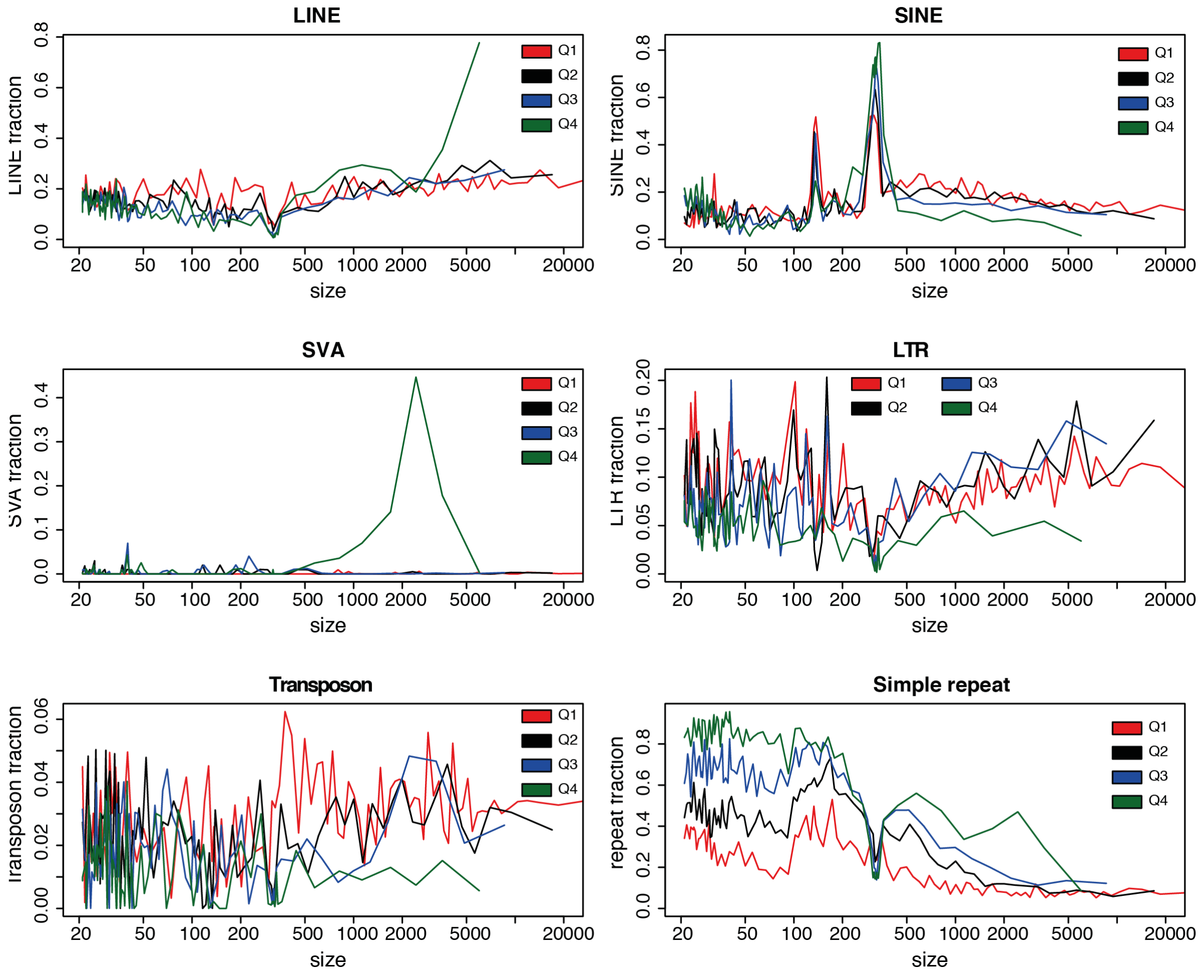

Supplementary Figure 1. Repeat content of deletions identified in the GoNL dataset. Deletions were grouped in quartiles (Q1-Q4) based on their allele frequency with the GoNL sample set. For each of the quartiles the overlap with six different repeat features was calculated (based on Ensembl release 75). The horizontal axis displays the mean size of deletions for each window of 100 deletions, ranked by deletion size. The vertical axis represents proportions (% basepairs) of repeat classes per window of 100 deletions.

1.2 Supplementary Figure 2

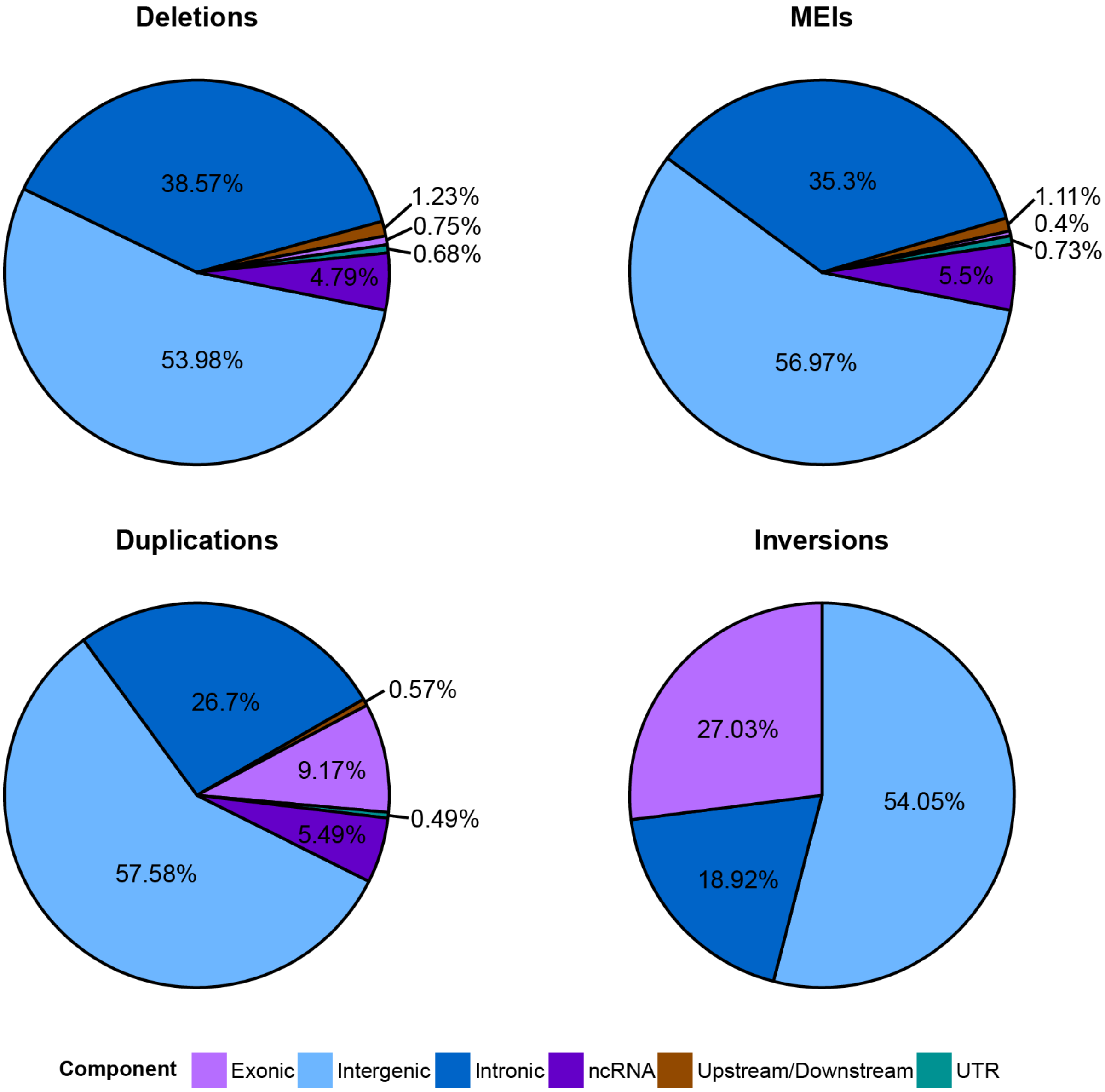

Supplementary Figure 2. The gene components affected by different types of structural variations in the consensus set. The majority of deletions (A), mobile element insertions (B), duplications (C) and inversions (D) are intergenic. The remainder of the deletions affected intronic sequence (38.6%) and less than 5% overlap an exon (RefSeq). A smaller minority overlap ncRNA, UTRs or are directly upstream or downstream of a known gene. Likewise 35.3% of MEIs overlap intronic sequence and 5.5% overlap exonic regions in the genome. In comparison a smaller proportion of duplications and inversions overlap intronic regions, 26.7% and 18.9% and larger proportion of duplications and inversions overlap exonic regions, 9.2% and 27% respectively.

1.3 Supplementary Figure 3

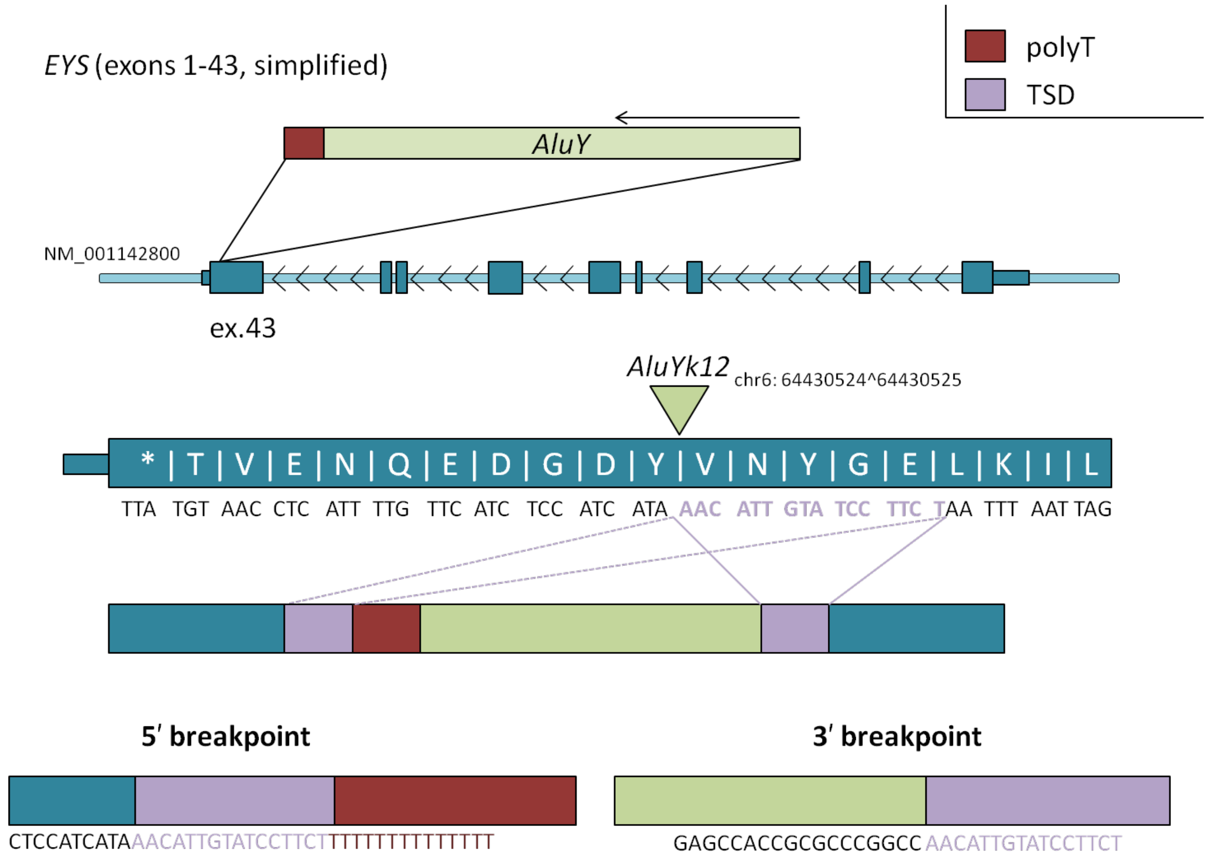

Supplementary Figure 3. A rare *ALUYk12* non-reference insertion identified in one single family at chr6:64430524, affecting exon 43 of *EYS* and resulting in a premature stop. The mobile element insertion contains a 16bp target site duplication.

1.4 Supplementary Figure 4

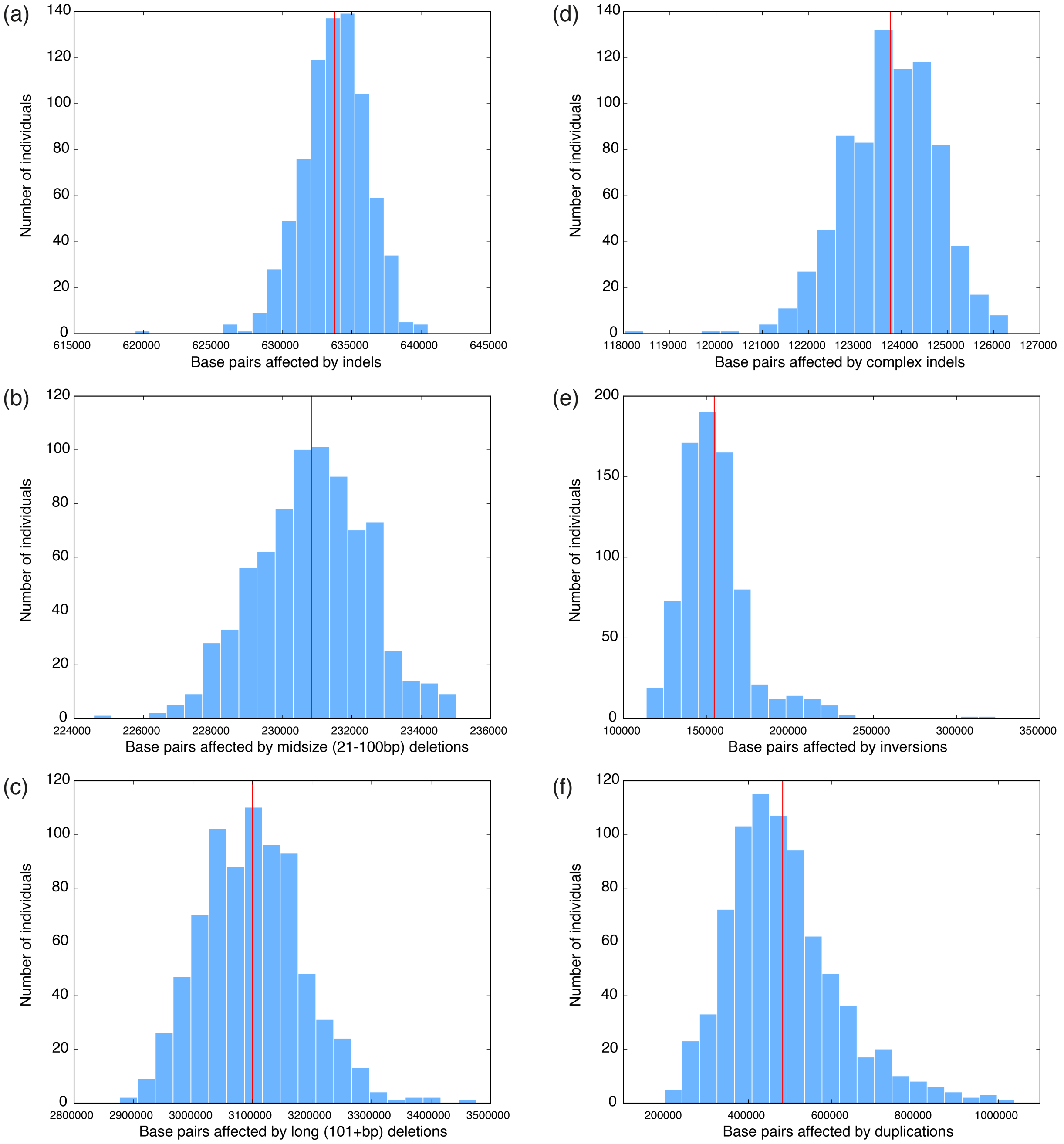

Supplementary Figure 4. Histograms showing the distribution of the number of affected base pairs per haplotype. For each individual, the number of affected base pairs was averaged over the two haplotypes, i.e. a heterozygous genotype contributes 0.5*length, where a homozygous genotype contributes 1*length. For missing genotypes, a contribution of AF*length is assumed. Red vertical lines indicate the average.

1.5 Supplementary Figure 5

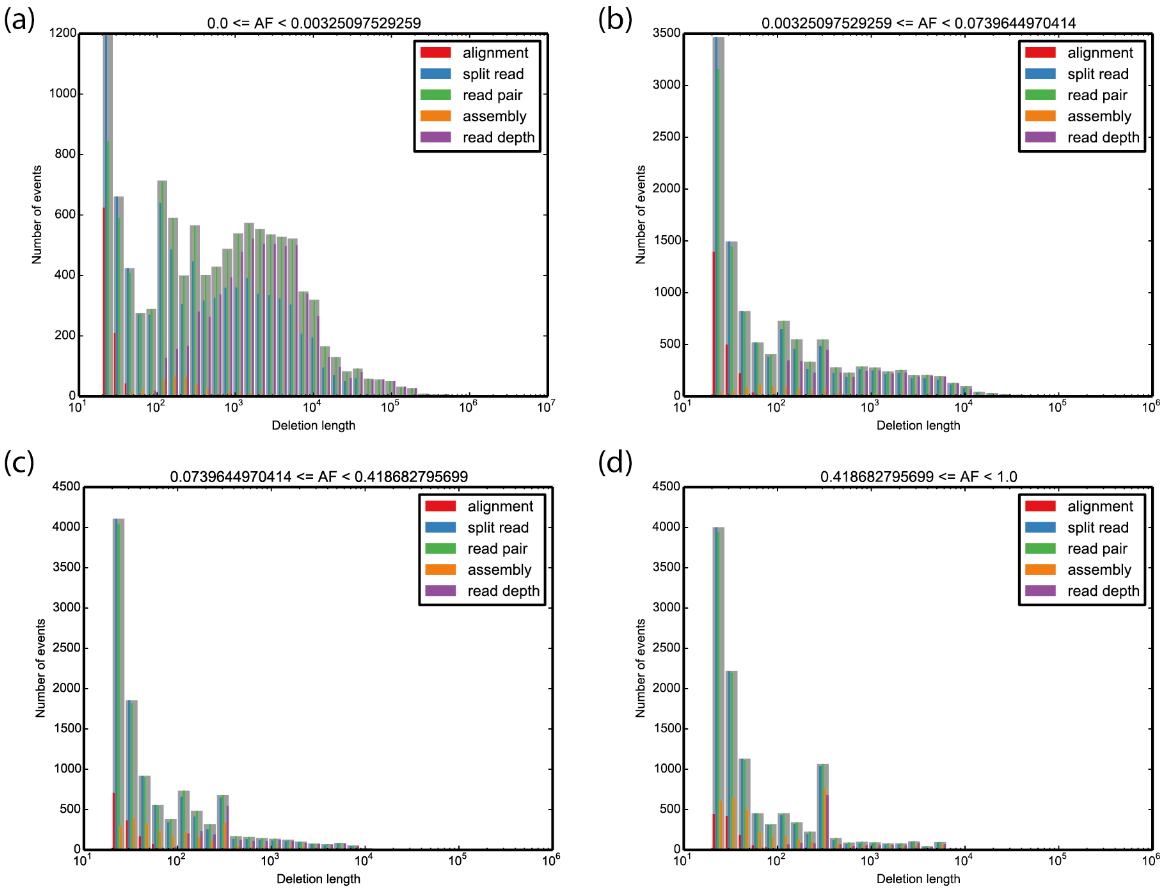

Supplementary Figure 5. Barplots showing deletion frequencies versus deletion size. Deletions have been stratified into four bins based on AF quartiles (panels a-d). Each plot shows the length distribution in the given AF range. Furthermore, thin bars indicate the approaches that have contributed to the discovery of the deletions in the respective size and AF range. The same deletion can have been discovered by multiple approaches, so each five thin bars can add up to more than the enclosing grey bar.

1.6 Supplementary Figure 6

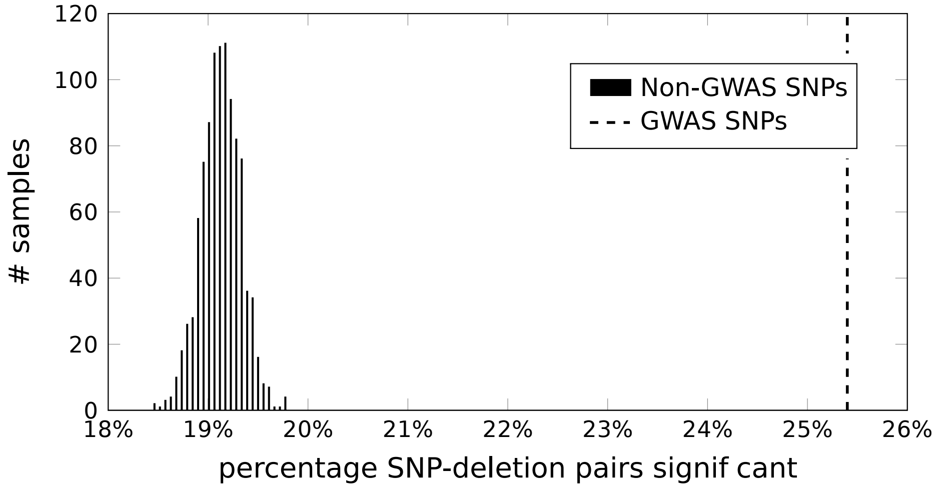

Supplementary Figure 6. The percentage of SNP-deletion pairs deemed statistically significant. The distribution represents the percentages found for 10^3 matched non-GWAS SNP-deletion samples (mean = 19.14%, sd = 0.20). The dashed line at 25.40% is the percentage found for the GWAS SNP-deletion pairs.

1.7 Supplementary Figure 7

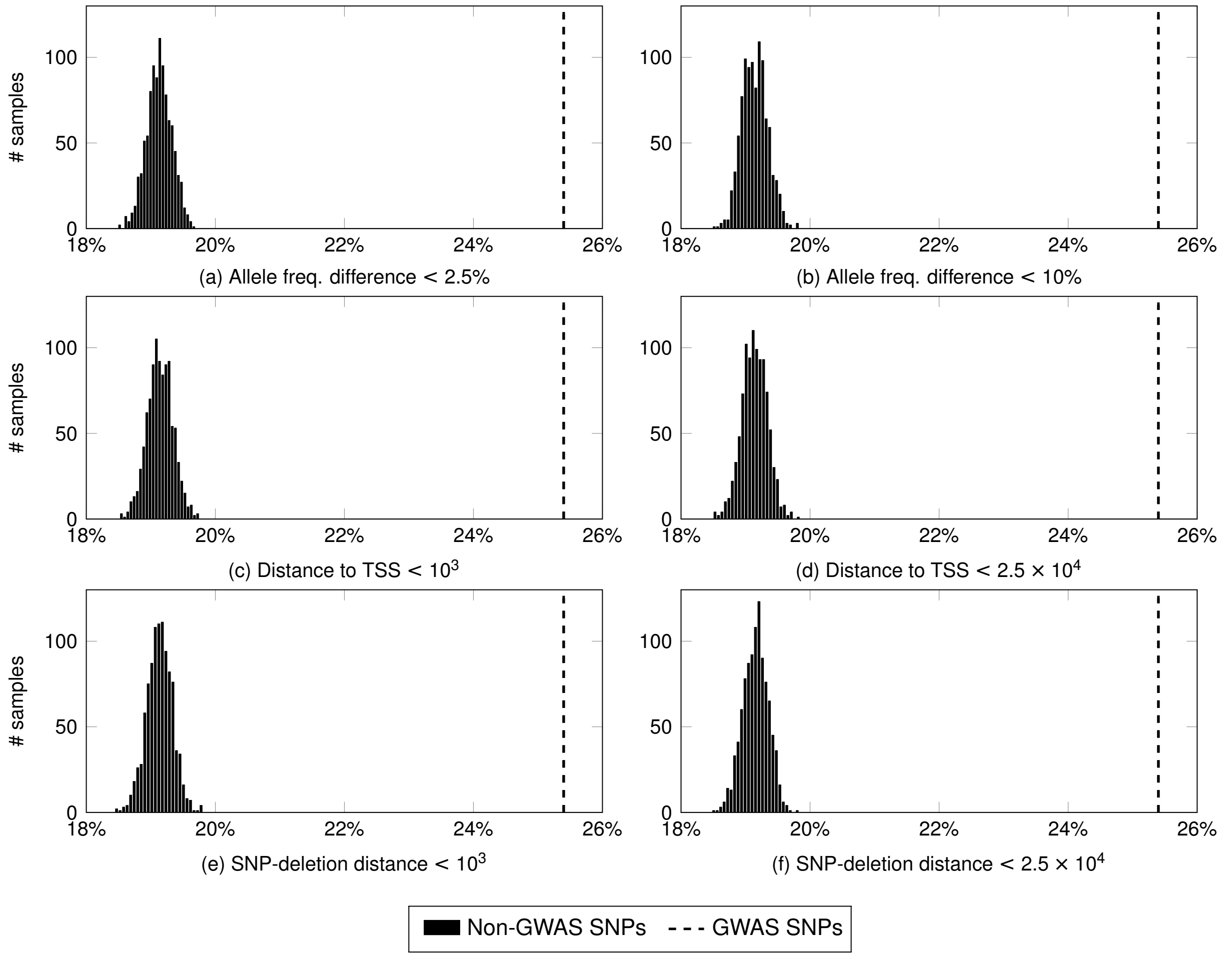

Supplementary Figure 7. The percentage of SNP-deletion pairs deemed statistically significant when varying the allele frequency and distance thresholds used for determining whether a non-GWAS SNP-deletion pair is similar to a GWAS SNP-deletion pair. The allele frequency threshold and the threshold for the distance to the nearest TSS and the distance between SNP and deletion are by default set to 5% and 10^4, respectively. Figure (a) and (b): only the allele frequency threshold is varied and set to 2.5% and 10% respectively. Figure (c) and (d): only the TSS threshold is varied and set to 10^3 and 2.5x10^4. Figure (e) and (f): only the threshold related to the distance between SNP and deletion is varied and set to 10^3 and 2.5x10^4.

1.8 Supplementary Figure 8

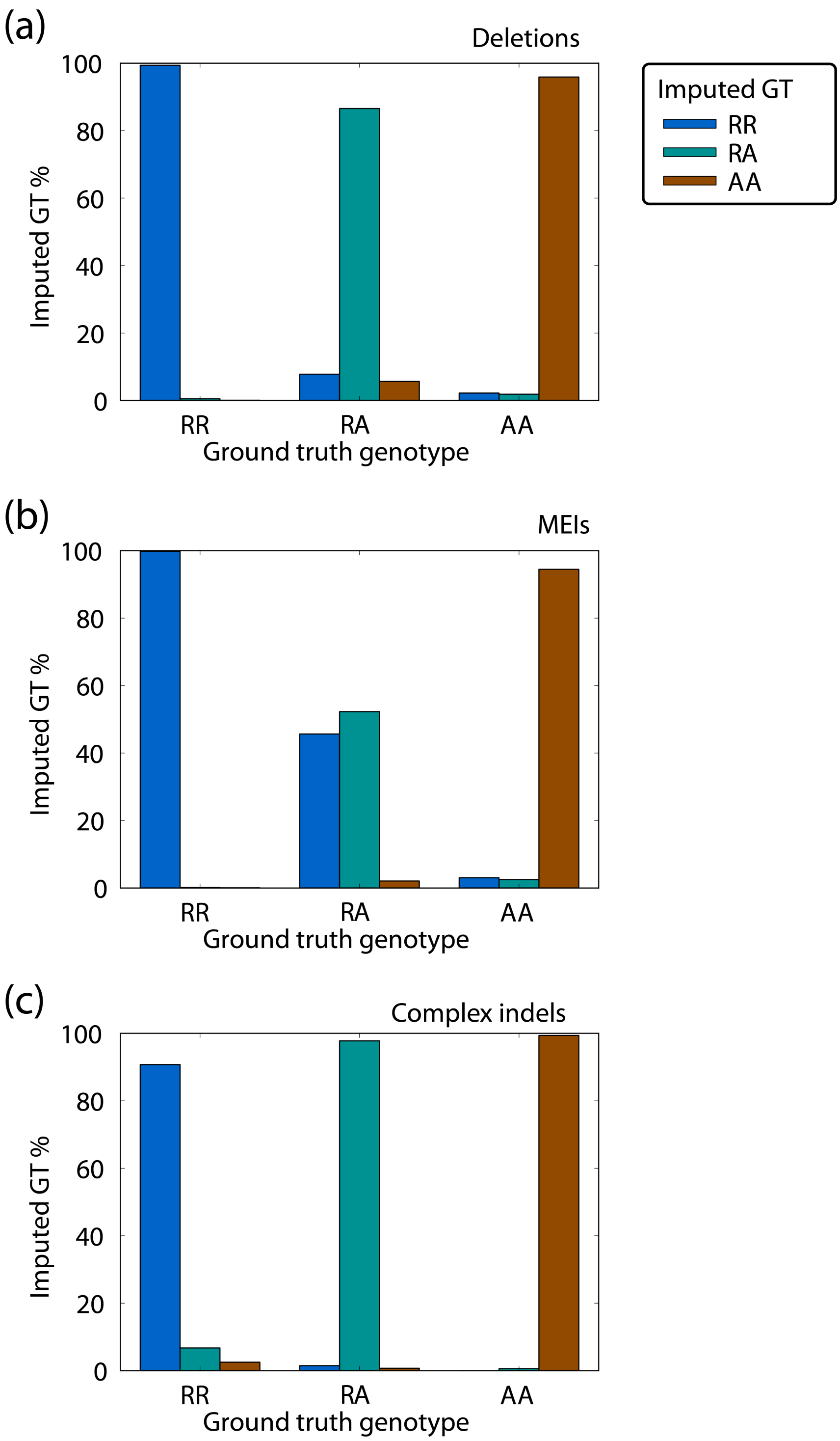

Supplementary Figure 8. Distribution of imputed genotypes per ground truth genotype and per SV type, where RR, RA, and AA are short for “homozygous in the reference allele”, “heterozygous”, and “homozygous in the alternative allele”. Imputed genotypes have been filtered at a GL threshold of c=0.95.

1.9 Supplementary Figure 9

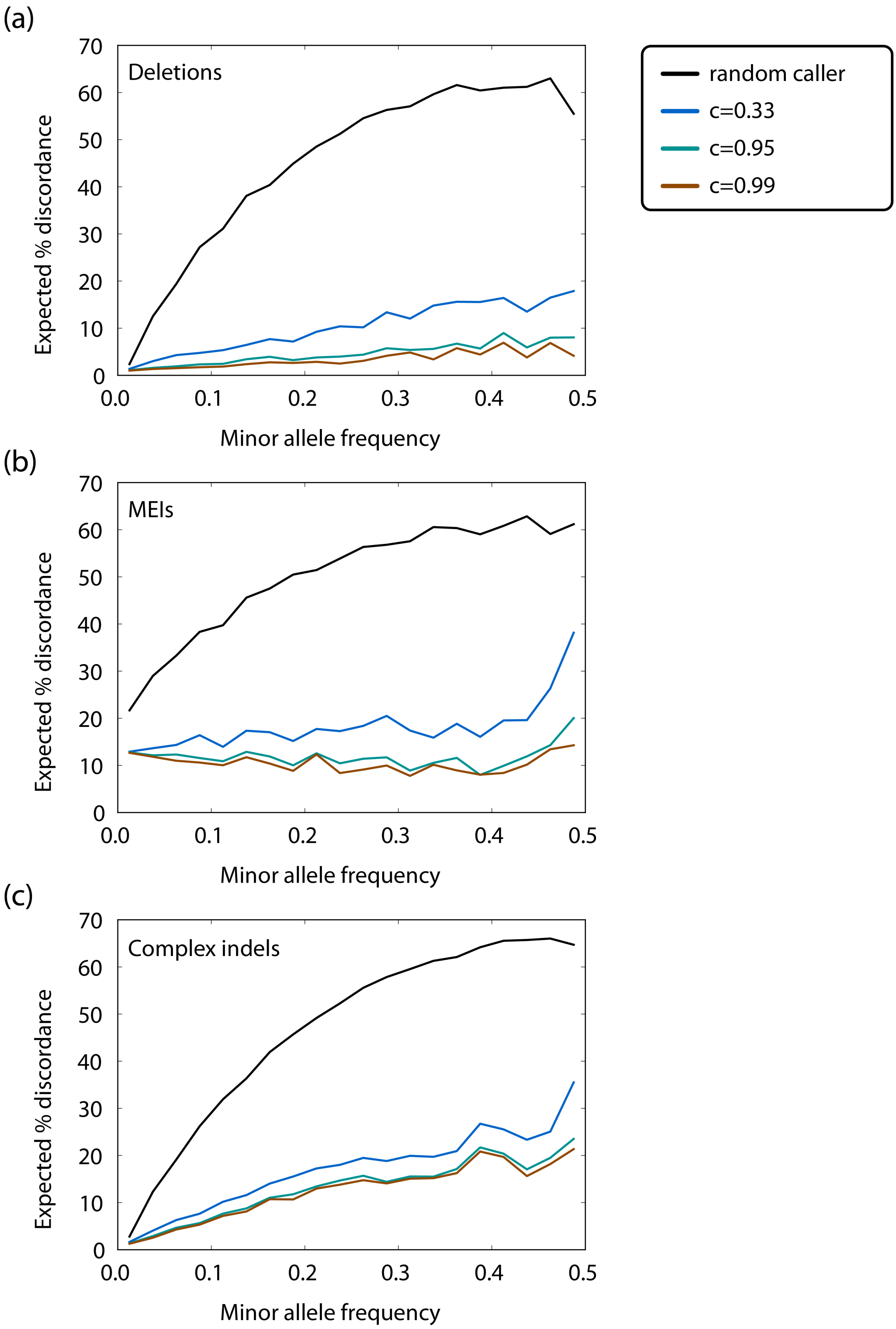

Supplementary Figure 9. Expected discordance rate stratified by minor allele frequency for different calling thresholds (c = 0.33, c = 0.95, and c = 0.99). Also the results of a panel-based guess, i.e. of a base-line random caller, are shown (black).

1.10 Supplementary Figure 10

(a)

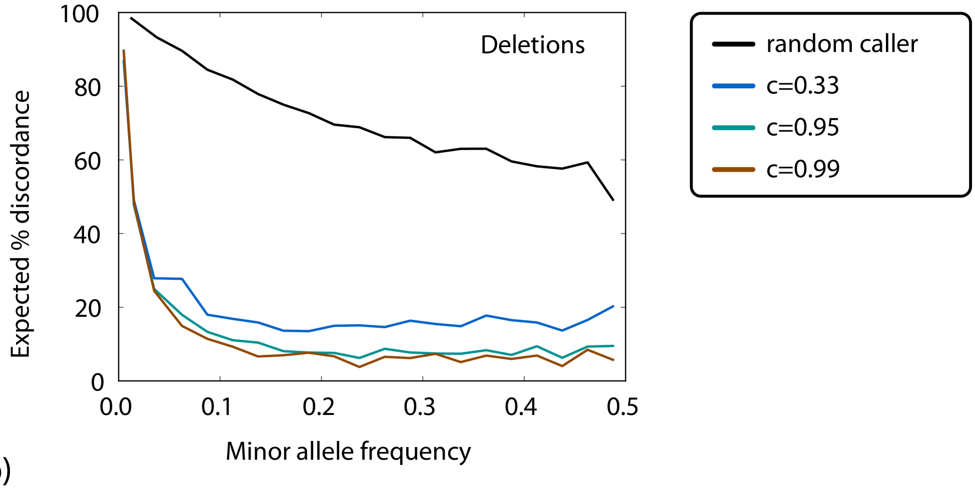

(b)

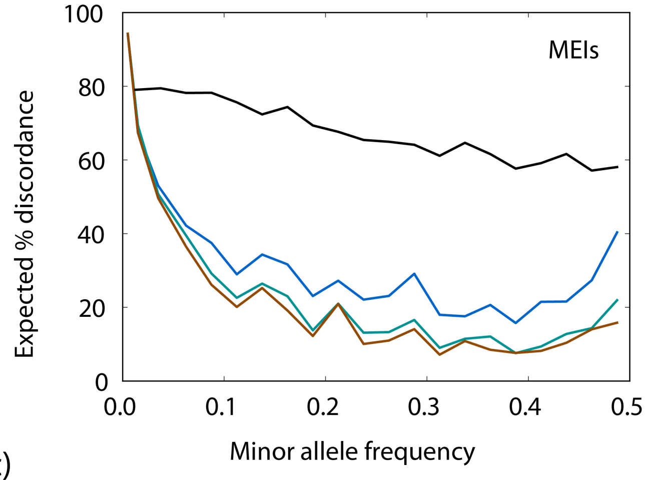

(c)

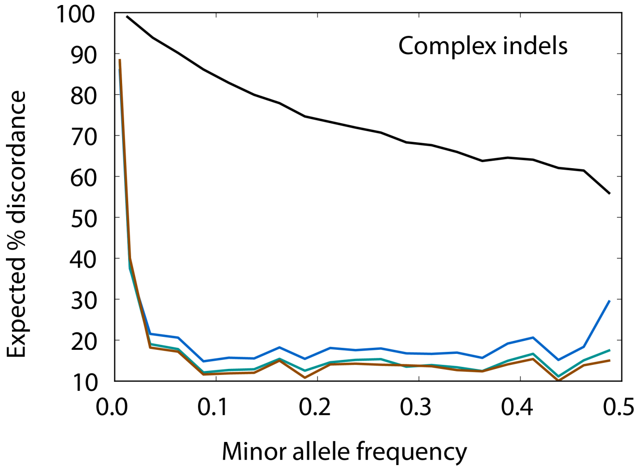

Supplementary Figure 10. Expected discordance rate stratified by minor allele frequency for different calling thresholds (c = 0.33, c = 0.95, and c = 0.99), when only gold standard genotypes containing the rare allele are considered. Also the results of a panel-based guess, i.e. of a base-line random caller, are shown (black).

1.11 Supplementary Figure 11

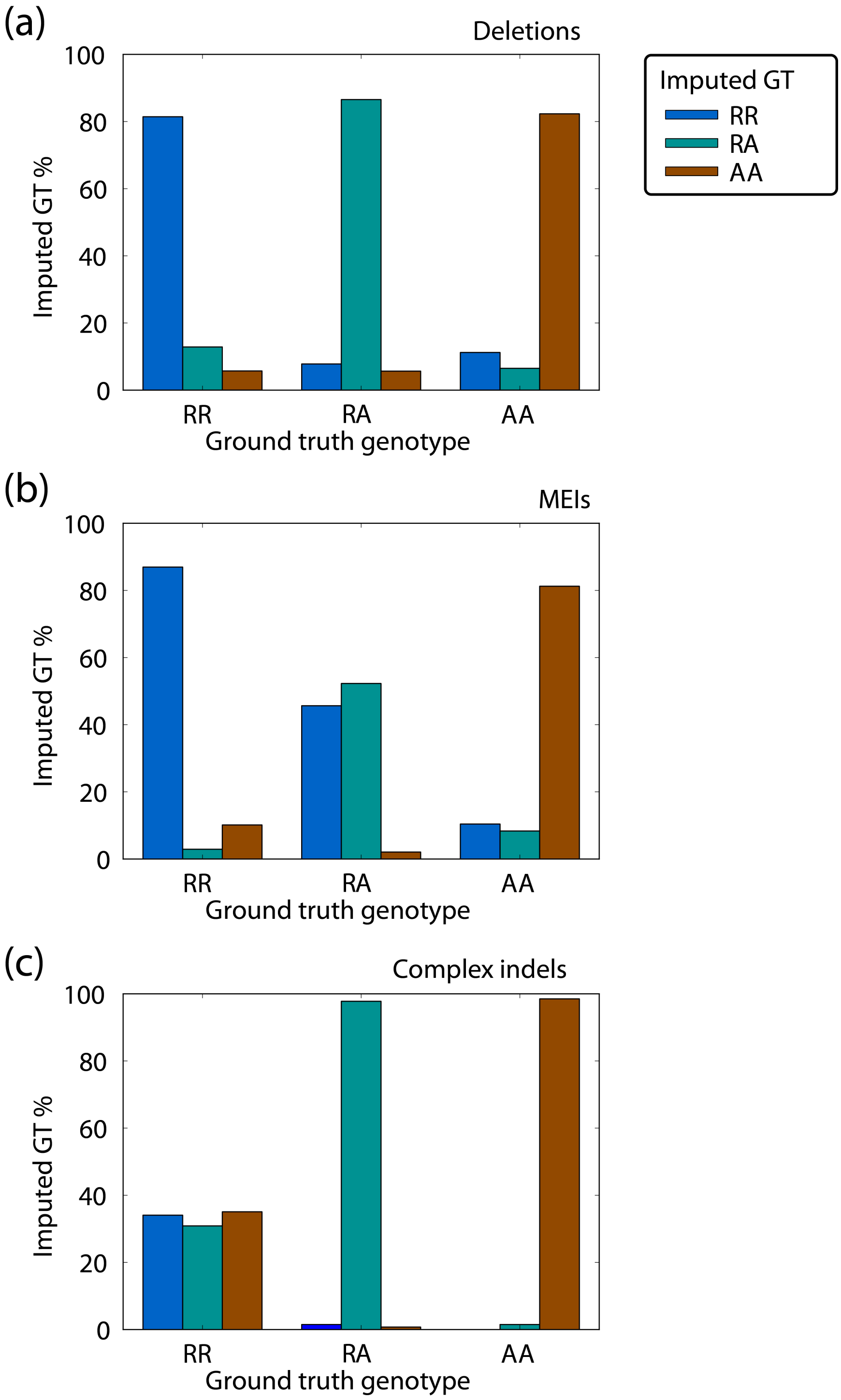

Supplementary Figure 11. Distribution of imputed genotypes per 'ground truth' genotype and per SV type when only genotypes for which the gold standard genotype contains the rare allele are considered. Note that either the reference or the alternative allele can be the rare(r) allele. RR, RA, and AA are short for "homozygous in the reference allele", "heterozygous", and "homozygous in the alternative allele". Imputed genotypes have been filtered at a GL threshold of c=0.95.

2 Supplementary Tables

2.1 Supplementary Table 1

Merging strategy for different detection algorithms to create the consensus set of structural variants.

Algorithm	Approach	Priority	Dels	Dups	Tandem Duplications	Ins	Trans	Inv	MEI
GATK UG	Split Read	1	Yes	Yes	N/A	Yes	N/A	N/A	N/A
GATK HC	Gapped Read	2	Yes	N/A	N/A	Yes	N/A	N/A	N/A
Pindel	Split Read	3	Yes	Yes	N/A	Yes	N/A	Yes	N/A
MATE-Clever	Split Read / Read Pair	4	Yes	Yes	N/A	N/A	N/A	N/A	N/A
SOAPdeNovo	De Novo Assembly	5	Yes	Yes	N/A	Yes	Yes	N/A	N/A
123SV	Read Pair	6	Yes	Yes	Yes	Yes	Yes	Yes	N/A
Breakdancer	Read Pair	7	Yes	Yes	N/A	Yes	Yes	Yes	N/A
GenomeSTRiP	Read Depth / Read Pair	8	Yes	Yes	N/A	N/A	N/A	N/A	N/A
DWACSeq	Read Depth	9	Yes	Yes	Yes	N/A	N/A	N/A	N/A
CNVnator	Read Depth / Read Pair	10	Yes	Yes	Yes	N/A	N/A	N/A	N/A
Façade	Read Depth	11	Yes	Yes	N/A	N/A	N/A	N/A	N/A
Mobster	Split Read / Read Pair	N/A	N/A	N/A	N/A	N/A	N/A	N/A	Yes

2.2 Supplementary Table 2

Deletion events in transmission disequilibrium

#Minimum total transmitted alleles = 20, two sided binomial test

Position	Length	Trans-mitted REF	Trans-mitted Alt	Chi Square	ChiSquare Pvalue	Exact binom.p	binom.test adjusted.p fdr	Chisquare test adjusted p.fdr
18:67949598	325	97	42	21.76259	3.09E-06	3.51E-06	0.0347927	0.030608
19:47993708	56	0	22	22	2.73E-06	4.77E-07	0.0094466	0.030608
11:128042965	327	55	17	20.05556	7.52E-06	8.14E-06	0.0366987	0.037245
7:147355052	313	57	18	20.28	6.69E-06	7.24E-06	0.0366987	0.037245
1:241899141	40	59	20	19.25316	1.14E-05	1.30E-05	0.0366987	0.037641
21:43350116	3369	49	14	19.44444	1.04E-05	1.11E-05	0.0366987	0.037641
19:54555525	5117	24	2	18.61538	1.60E-05	1.05E-05	0.0366987	0.045282
5:2907781	230	40	10	18	2.21E-05	2.39E-05	0.0438806	0.04715
5:82063426	2727	30	5	17.85714	2.38E-05	2.24E-05	0.0438806	0.04715
6:136596839	141	49	15	18.0625	2.14E-05	2.44E-05	0.0438806	0.04715
6:29815075	496	1	20	17.19048	3.38E-05	2.10E-05	0.0438806	0.051509

2.3 Supplementary Table 3

Overview of classes of novel genomic segments identified in the GoNL dataset.

		GoNL new segments			Matching to GRCh38, megabases				GRCh38 in GoNL, %
Set	Samples containing segments	Count	Total Mb (with gaps)	Total Mb (no gaps)	Chromosomes	Alternative haplo-types	Un-mapped/ not localized	Decoy (hg38d1)	
Fixed (>95% samples)	731-769	1517	2.486	2.379	1.084	0.013	0.102	0.149	56.66
Common (5-95% samples)	39-730	7236	4.32	4.102	0.977	0.206	0.031	0.614	44.56
Rare (<5% samples)	1-38	2250	0.761	0.714	0.187	0.012	0.001	0.04	33.61
Males	Males	235	0.216	0.215	0.099	0	0	0.009	50.23
Herpes-virus (CIHHV6)	4 (HHV6b in gonl-54b,c; HHV6a in gonl-115b,c)	112	0.207	0.163	0	0	0	0	0.00
**Total internal**		**11350**	**7.99**	**7.573**	**2.347**	**0.231**	**0.134**	**0.812**	43.48
Bacteria-like[*]	12(gonl-37a,b; gonl-84a,b; gonl-122a,b; gonl-141a,b; gonl-193a,b; gonl-217a,b)	3439	13.962	13.067	0	0	0	0	0.00
Mouse-like[**]	From 7 families (gonl-4; gonl-9; gonl-149; gonl-152; gonl-167; gonl-221; gonl-242)	38	0.015	0.015	0	0	0	0	0.00
Rat-like	gonl-113a	23	0.014	0.014	0	0	0	0	0.00
Plant[***]	4 (gonl-143a,c; gonl-187b,c)	159	0.166	0.165	0	0	0	0	0.00
Plant2[****]	1 (gonl-225b)	89	0.035	0.028	0	0	0	0	0.00
**Total external**		**3748**	**14.192**	**13.289**	**0**	**0**	**0**	**0**	0.00

[*]Homology to *Achromobacter* and *Burkholderia* DNA, samples from a single biobank

[**]Samples from single biobank

[***]Homology to Lactuca DNA

[****]Homology to Violet DNA

2.4 Supplementary Table 4

Imputation results using GoNL panel (only SVs overlapping between GoNL and 1000 genomes projects were used): % discordance, % missing and total number of calls per SV type for four calling thresholds (c = 0.33, c = 0.95, c = 0.99 and c = 0.999), averaged over the two independent study samples. The right-hand side of the table shows the results when evaluating minor allele genotypes only. For inversions, transpositions and duplications we do not have sufficient calls to consider the results for minor allele genotypes only.

	All genotypes			Minor allele genotypes only		
	% disc.	% missing	# calls	% disc.	% missing	# calls
Deletions						
c = 0.33	3.2	0.0	3801	12.7	0.0	629
c = 0.95	1.2	6.3	3678	5.5	20.6	494
c = 0.99	0.9	9.4	3555	4.5	28.5	450
c = 0.999	0.6	14.9	3331	3.5	40.8	376
Duplications						
c = 0.33	3.7	0.0	40	-	-	1
c = 0.95	0.1	6.1	38	-	-	0
c = 0.99	0.0	7.3	38	-	-	0
c = 0.999	0.0	13.4	35	-	-	0
Inversions						
c = 0.33	12.6	0.0	28	-	-	3
c = 0.95	11.4	7.0	26	-	-	2
c = 0.99	8.3	15.5	24	-	-	1
c = 0.999	6.7	19.7	22	-	-	1
MEI						
c = 0.33	6.0	0.0	3889	13.6	0.0	827
c = 0.95	3.2	7.1	3651	7.5	19.4	660
c = 0.99	2.7	10.4	3226	6.1	25.8	610
c = 0.999	2.3	25.9	3305	4.9	35.2	537

2.5 Supplementary Table 5

Imputation results using 1000 Genomes panel (only SVs overlapping between GoNL and 1000 genomes projects were used): % discordance, % missing and total number of calls per SV type for four calling thresholds (c = 0.33, c = 0.95, c = 0.99 and c = 0.999), averaged over the two independent study samples. The right-hand side of the table shows the results when evaluating minor allele genotypes only (based on allele frequencies in 1000 Genomes data). For inversions, transpositions and duplications we do not have sufficiently many calls to consider the results for minor allele genotypes only.

	All genotypes			Minor allele genotypes only		
	% disc.	% missing	# calls	% disc.	% missing	# calls
Deletions						
c = 0.33	5.8	0.0	3910	18.4	0.0	660
c = 0.95	2.7	8.7	3571	12.9	33.5	520
c = 0.99	2.2	11.9	3444	11.5	41.3	474
c = 0.999	1.8	15.5	3305	11.0	48.1	432
Duplications						
c = 0.33	0.0	0.0	37	-	-	0
c = 0.95	0.0	0.0	37	-	-	0
c = 0.99	0.0	0.0	37	-	-	0
c = 0.999	0.0	1.3	36	-	-	0
Inversions						
c = 0.33	14.2	0.0	28	-	-	1
c = 0.95	8.3	13.9	24	-	-	0
c = 0.99	8.5	17.0	23	-	-	0
c = 0.999	8.9	19.7	22	-	-	0
MEI						
c = 0.33	11.1	0.0	3928	39.1	0.0	876
c = 0.95	5.2	14.5	3360	30.3	54.6	408
c = 0.99	4.3	17.9	3226	29.5	63.7	328
c = 0.999	3.7	21.4	3089	28.9	70.9	264

3 Supplementary Methods

3.1 Indel and structural variant calling

Variant identification was performed using a combination of 12 different algorithms, which represent 5 major approaches for indel and structural variation (SV) discovery: (i) gapped reads, (ii) split-read, (iii) read pair, (iv) read depth and (v) *de novo* assembly (**Figure 1**) [1]. We have defined indels as insertions and deletions with a size smaller than 21bp and SVs comprise all types of variants larger than 20bp. The subsections below describe details for each of the calling algorithms. Part of the variant calling has been described previously, but these parts are repeated here for completeness [1,2].

3.1.1 Pindel

We applied Pindel (https://github.com/genome/pindel version 0.2.4t) [3] on the complete GoNL alignment BAM files and the calculation was carried out on the Dutch Life Science Grid (https://grid.sara.nl/wiki/index.php/Life_Science_Grid). We used the default parameter set for the calculation. For this analysis, all chromosomes were split into bins of 20 MB, with an overlap of 0.1 MB, resulting in 113 genomic regions, spread over 75,000 files. The data itself was filtered to only include reads of which one end could not be mapped to the reference genome, significantly reducing the file size for each region and resulting in less transfer overhead. In the second step of the analysis, one region was selected and analyzed across all samples. For each analysis 8 compute cores were used, consuming 5000 core hours in total. The confident variants are those observed in one child with at least 2 supporting reads and which also appeared in at least one of the parents.

3.1.2 GATK Unified Genotyper

We used the GATK UnifiedGenotyper (UG; https://software.broadinstitute.org/gatk/) v2.1.8 for indel discovery and calculation of genotype likelihoods, running it on 5 Mb regions with 100 bp overlap on each side. The overlap ensured that an indel at the fringe of the 5 Mb

region would be correctly called. In order to separate biallelic sites from multi-allelic ones, we used the GATK UG multi-allelic model for indel calling.

Only bi-allelic ones were kept for further processing and downstream analyses. We only reported indels with a Phred quality score > Q10.

We adopted a filtering strategy by using GATK VQSR on our initial calls. We used the following specifications:

a) Training sets

i. Mills-Devine 1KG gold standard indel set [4]

b) Features

i. Quality / Depth

ii. Fisher's test on strand bias

iii. Haplotype score

iv. Read position rank sum test

v. Inbreeding coefficient

3.1.3 GATK HaplotypeCaller

We used the GATK HaplotypeCaller (https://software.broadinstitute.org/gatk/) to discover and genotype the union of regions previously called as putative indels in GoNL [2], raw indel calls as well as Pindel calls [3], including 1kbp flanking each variant. We filtered these calls using GATK VariantQualityScoreRecalibration (VQSR) using the following parameters:

a) Training sets

i. Mills-Devine 1KG gold standard indel set [4]

b) Features

i. Quality / Depth

ii. Fisher's test on strand bias

iii. Haplotype score

iv. Read position rank sum test

v. Inbreeding coefficient

We genotyped all autosomal calls passing VQSR with GATK PhaseByTransmission using a mutation prior of 10^{-4} to obtain sensitive trio-aware genotypes.

3.1.4 1-2-3-SV

We used version 0.9 of the 1-2-3-SV software package (http://tools.genomes.nl/123sv.html) [5] for population-based calling of deletions, inversions, intra- and inter-chromosomal structural variants. The analysis was performed across the complete set of 2,238 libraries. We considered only uniquely mapped reads (BWA X0 flag = 1) that do not have a secondary hit (X1 flag = 0). A read pair was considered to be discordant if the two reads in a pair map to different chromosomes, display incorrect mapping orientation or exhibited too short (below 0.1 percentile) or too long (above 99.9 percentile of fragment size distribution characteristic for a given library) distance of the mapping positions. We only retained clusters supported by 4 or more discordant and independent (not PCR duplicates) read pairs.

3.1.5 Breakdancer

We used BreakDancer (http://breakdancer.sourceforge.net) version 1.1 with parameters -r 5 -c 7 [6]. The rest of the parameters were set to default). Calling was performed per family. Calls from different families that have overlapping confidence intervals of both breakpoints were merged in an iterative fashion, using a custom Perl script. Clusters supported by at least five read pairs were retained for further analysis.

3.1.6 DWAC-Seq

Version 0.7 of DWAC-Seq (http://tools.genomes.nl/dwac-seq.html) was used to call segmental copy-number changes. A BAM file representing a common GoNL reference set was constructed by taking 0.13% (1/769) reads from each of the individual samples and merging them into a single file. Each GoNL sample was then compared against this common reference using dynamic windows that overlap 250 unambiguously mapped sequencing

reads (BWA flag X0=1). We filtered out CNV candidates that showed a copy-number change of less than 30%. CNV calls from different families with 80% reciprocal overlap were merged together in an iterative manner using a custom Perl script.

3.1.7 CNVnator

The CNVnator (http://sv.gersteinlab.org/cnvnator/) v0.2.2 read-depth algorithm was used for calling CNVs [7]. The read-depth signal consists of readcounts in non-overlapping genomic bins of equal size. Subsequently, the read-depth signal is then segmented into regions with different copy numbers. CNVs calls are predicted on the basis of statistical significance tests performed on the segmented regions.

To determine the optimal bin size for our dataset, we analyzed a quartet with MZ twins using CNVnator with nine different bin sizes (ranging from 100 - 500 bp, with an increment of 50 bp). We found that a binsize of 500 bp resulted in the highest concordance between the MZ twin samples and therefore used this setting for the full data set.

We applied a GC correction and included CNVs in the final call on the basis of the following criteria: (i) $p < 0.05$ (corrected for multiple testing), (ii) the proportion of reads in the CNV that map to > 1 location does not exceed 50%, and (iii) the CNV does not overlap with an assembly gap of the reference genome or centromere.

3.1.8 FACADE

Short reads were mapped using *mrsFAST* to a repeat-masked reference (hg19) [8]. A correction for GC bias was applied to the raw read depth. An initial quality control step involved calculation of the correlation (R^2) between copy numbers and coverage at known loci with a fixed genotype. One sample with $R^2 < 0.85$ was withdrawn from further analysis. With read depth information, we could predict the copy number of a gene or a segment of the genome. As a second quality control step, we performed copy number calls with 3 kb windows across the whole genome. Samples that had > 120,000 windows with a copy

number of 2 were discarded, leading to the exclusion of 30 samples. In total, 224 families including all 19 quartets met the criteria and were used for variant calling.

To discover CNV events, we first estimated copy numbers across the whole genome from read-depth analysis using windows of 1kb of unique sequence. Then, copy number $\log_2$ ratios between offspring and parents as well as all samples versus the population median were calculated. All pairwise $\log_2$ ratios were segmented using the previously published method FACADE to produce the initial call set [8]. To account for variability in call boundaries with events encompassing a small number of windows of variable genomic size, we applied a Multiscale French Tophat wavelet to refine the breakpoints for events between 2 and 7 windows in size. Each call was classified into four classes: (i) *de novo*, (ii) assortment, (iii) inherited, and (iv) common to the family. To filter out low-confident calls, only calls spanning ≥ 2 windows and with a log ratio greater than 0.31 or less than -0.25 for duplications and deletions, respectively, were retained.

3.1.9 Mate-Clever

For deletion discovery, we ran the discovery part of MATE-CLEVER [9], with minor modifications that account for different insert size distributions in different libraries.

MATE-CLEVER is an integrated approach. Its major purpose in the frame of the project is to discover deletions of size 30--100 bp (sometimes termed the "twilight zone of NGS indels").

It incorporates CLEVER [10], as an internal segment size based approach and LASER [9], as a split-read aligner.

MATE-CLEVER uses CLEVER in a first step, in order to discover deletions of size 30--100 bp at extremely high sensitivity, and uses LASER in a second step, in order to refine the breakpoint annotations made by CLEVER. It also uses several auxiliary tools, as described below. In the following, we provide the full description of details and commands, by which to reproduce MATE-CLEVER's callset.

To run the CLEVER-based deletion discovery pipeline, revision 3097f2 from the git repository

at https://bitbucket.org/tobiasmarschall/clever-toolkit has been used. It includes CLEVER, LASER, and several auxiliary tools described below. To run (and parallelize) the below pipeline, we used the Python-based workflow engine Snakemake [11].

CLEVER was run on all individuals separately. In detail, that means the following steps were performed:

1. We used tool \texttt{bwabam-to-alignment-priors} to extract prior alignment probabilities. The output was split by chromosome. Commandline: "bwabam-to-alignment-priors -m mean-and-sd.txt ref.fasta input.bam | split-priors-by-chromosome -z -s input.bam priors". For each chromosome and each sample in the input, one gzipped file prefixed with "priors" is created. All alternative alignments provided by BWA through XA tags are used. Furthermore, this step generates robust estimates of insert size mean and standard deviation, which are stored in the file mean-and-sd.txt.
2. All files with alignment probabilities are sorted by genomic position by means of a standard unix sort (sort -g -k7).
3. The CLEVER core engine is run on each of these files. Command line: "zcat input.probabilities.gz | clever -c 150 -v > clever.output". The option "-c 150" instructs CLEVER to limit local coverage to 150 to avoid long runtimes for regions with excess coverage (due to repeats).
4. For each individual, all CLEVER output files for the chromosomes are concatenated and postprocessed. Commandline: "postprocess-predictions -d5 --only-del concatenated-clever-output.txt <mean>", where <mean> is the mean insert size as estimated in Step 1.1. As per option --only-del, only deletions are extracted and processed further. Option \texttt{-d 5} is used to only retain deletion predictions supported by significant cliques with at least 5 alignment pairs.

LASER was run on all regions with putative deletions as identified by CLEVER in Step 1:

1. To generate high-quality background distributions for insert size, insertion size, and

deletion size from uniquely mapping reads, LASER was used to realign reads from randomly chosen regions. To this end, 5000 regions of length 10000 were sampled uniformly at random. Reads mapped to these regions by BWA were extracted and remapped using LASER as described previously [9]. The following command line was used for the LASER core step: "join-split-reads -XIS -A14 --anchor_distance 2000 --max_span 2000 -L insert-size.dist -R insertion-size.dist -D deletion-size.dist ref.fasta input.1.fastq.gz input.2.fastq.gz". For all individuals/families, the same set of random regions was used.

2. For every family, CLEVER deletion predictions for all individuals are pooled and a set of regions of these deletions plus 500 bp up- and downstream of each deletion is created.
3. For all individuals, reads aligned to the selected regions by BWA were extracted. When only one read in a pair was successfully mapped by BWA, the unmapped read was also included. The following parameters were used: "join-split-reads -XIS -A14 --anchor_distance 2000 --max_span 50000 -P putative-indels --snp putative-snps ref.fasta input.1.fastq.gz input.2.fastq.gz > output.bam". In this mode, LASER writes lists of putative SNPs and indels to the given filenames. Each of these comes with an expected support, i.e. the expected number of reads giving evidence for that particular SNP/indel.
4. Evidence from all individuals of the considered family is pooled, i.e., the expected support is added over all family members for all indels reported by LASER. Further filtering is done as follows. We retained all deletion candidates that have a total expected support of at least 0.5, are at least 10 bp long, and agree with a deletion prediction made by CLEVER. Here, we deem two putative deletions "agreeing" if their center points are at most 100 bp apart and the length difference is at most 20 bp. The resulting set of deletion candidates was used for recalibration of alignments produced by LASER in the next step.
5. The scores of all alignment pairs were recalibrated as follows. Phred-scaled insertion

and deletion probabilities were set according to the empiric distribution obtained in Step 2.1. Deletions in the candidate set generated in Step 2.4 incur a constant (length-independent) alignment cost of 1. This upweights alignments that support deletions that have also been reported by CLEVER in Step 1 and are thus in line with read pair evidence. For each read pair the alignment pair with the highest probability is reported as primary alignment. All secondary alignments were discarded at this stage.

6. The number of LASER (primary) alignments supporting each deletion were extracted from the BAM files for further processing.

All deletions of CLEVER and LASER were merged. In this step, the whole GoNL population is considered simultaneously. We iterated over all putative deletions as reported by LASER sorted (decreasingly) by total support of primary alignments in the whole population.

1. For each individual, the set of CLEVER predictions was searched for deletions with a center distance of at most 100 and a length difference of at most 20 from the currently processed deletion. All CLEVER calls matching the current deletion are marked and ignored in subsequent iterations. This ensures that each CLEVER deletion is assigned to at most one LASER prediction, giving precedence to predictions common in the population.
2. If a deletion is supported by CLEVER with support of at least 5 and by at least one LASER (split) alignment, we report it to be present in the individual in question.
3. All deletions present in at least one individual are included in the final VCF file.

3.1.10 GenomeSTRiP

GenomeSTRiP (http://software.broadinstitute.org/software/genomestrip/) [12] v1.04.915 was used for deletion discovery. Each BAM file was pre-processed using the GenomeSTRiP preprocessing pipeline to produce GC-content, depth of coverage and insert size statistics files for GenomeSTRiP downstream modules. We performed deletion discovery using

standard settings (as for 1KG Phase 1) based on bins of 5 Mb with 100 kb overlap on all individuals concurrently to maximize sensitivity. Selection of deletions was done using the following criteria:

a) Fraction of deletion within alpha satellite < 90%

b) Mean number of aberrant read pairs per sample in which aberrant read pairs are found ≥ 1.1

c) Depth ratio of samples with aberrant readpairs < 0.63 (or < 0.8 if membership test p-value < 0.01)

d) Mean normalized read depth in samples contributing aberrant read pairs < 1.0

e) Depth p-value < 0.01 indicating sufficient power in the read depth tests

f) Read-pair coherence test statistic < 0.01

We genotyped all resulting candidate deletions and further filtered sites based on the samples' genotypes to exclude:

a) Sites with an inbreeding coefficient < -0.15

b) Likely duplicate sites overlapping other sites by more than 50% and with no distinguishing samples where the likelihood of discordant genotypes was > 99%

c) Likely non-variant sites without any confident (genotype quality ≥ Q13) non homozygous reference genotypes

d) Sites not clustering well based on the read-depth analysis

  i. Cluster separation (mean Mahalanobis distance between CN1 and CN2 clusters) < 2.0 for events up to 100kp, < 2.5 for larger events

  ii. Cluster means < 0.5 or > 2.0 times the normalized genome-wide read depth expectation

e) Sites with less than 200 bp of uniquely alignable sequence.

3.1.11 SOAPdenovo *de novo* assembly

We selected discordantly mapped and unmapped read pairs from each family and performed *de novo* assembly per family using SOAPdenovo2 [13], version r240 using kmer size 63 (http://soap.genomics.org.cn/soapdenovo.html). We then obtained family-specific contigs and assembled all family-specific contigs for the entire GoNL sample set together in a second round of *de novo* assembly. The resultant non-redundant contigs were compared against the GRCh37/hg19 genome reference using BLAT software. Alignments that consisted of two distantly mapped HSP segments of at least 150 base pairs that correspond to left and right part of a contig (with no more than 10 bp overlap at contig-level) were parsed by a custom script (available upon request) to define breakpoints of structural genome rearrangements.

3.1.12 Mobster

Non-reference mobile element insertions (MEIs) were identified using Mobster v0.1.6 with default parameters[14]. Analysis was run separately for each family. We only retained candidate MEIs supported by 5 or more reads.

The sensitivity for detection of MEIs by Mobster was separately tested using a set of 134 validated MEIs (127 *Alu*; 6 L1; 1 SVA) from 1000 Genomes sample NA12878 [15]. We subsampled the original data for NA12878 resulting in an average coverage of ~14X. By running Mobster on this subsampled dataset we could detect 104/134 (77.6%) validated MEIs based on a combination of both single and double cluster predictions.

3.1.13 Identification of novel genomic segments

Two types of variants called in section **3.1.11** were analysed separately: i) full contigs that exceed 150bp in size and did not map anywhere with >99% homology when mapped by NCBI BLAST against the GRCh37/hg19 assembly and ii) parts of contigs larger than 150 bases that did not map to any genomic location. We realigned the individual-specific sets of

discordant reads using these new segments as a reference sequence, in order to determine their presence/absence in the libraries of each individual.

We divided these new segments into two sets: i) potential contaminations (segments that appear in some of the libraries, but not in all libraries of the same individual) and ii) true new segments (**Supplementary Table 3**) that showed a consistent pattern of presence or absence across all libraries belonging to an individual. For the contamination dataset (3748 segments totaling 14.2 million base pairs), we defined five subsets on the basis of the pattern of appearance of these sequences in individual samples and their matches in the NCBI non-redundant DNA database. The dataset of true new segments was divided based on their population frequency (Fixed, > 95%; Common, 5-95%; rare < 5%), gender (Male-specific, >5% population) and a match to herpesvirus DNA (4 individuals from two families). Finally, we used NCBI BLAST to check if these segments were present in the most recent GRCh38/hg38 genome reference or a decoy dataset hg38d1. We required 99% identity in the alignment between assembled segment and latest genome reference to discard a segment as unreported by GRCh38.

3.1.14 Construction of simple indel set

A data set with simple indels (1-20 base pairs) was constructed by merging four individual callsets obtained by running GATK HaplotypeCaller, Pindel, Mate-Clever and SOAPdenovo assembly (as described in previous sections).

3.1.15 Construction of complex indel set

Genomic regions showing a high density of polymorphisms (distance between adjacent polymorphisms below 30 basepairs) were tested for being complex events or alleles that potentially appeared as part of the single mutational event, but called as separate adjacent events. GATK HaplotypeCaller was rerun for these regions using the ‘mergeVariantsViaLD’ option and non-simple indels were retained. Simple events that originally represented these complex events in the simple indel dataset were then excluded from the simple indel dataset.

3.2 Variant validation

We have previously described validation of 1-20bp indels (433 deletions, 407 insertions), 20-100bp deletions (96 variants) and 100+bp deletions (48 variants) based on the GoNL release 1 dataset [2].

Here, we describe an extension of these validations, which is based on the GoNL indel and SV call set released with this paper. For all validation rounds described below, we performed PCR amplification of the variant region or breakpoint junction regions (**Supplementary Data 1**). PCR primers were designed using primer3 software based on breakpoint coordinates. Subsequently, we performed sequencing of PCR products using Sanger sequencing or MiSeq (2*250bp). Sanger sequencing reads were mapped to the human reference genome (GRCh37) using BLAST/BLAT functionalities of the Ensembl genome browser (ensembl.org) or using NCBI BLAST. MiSeq reads were mapped to the GRCh37 reference using BWA. For each variant larger than 5bp a new contig was added to the reference sequence, based on exact variant breakpoint coordinates and a flanking genomic sequence on each side of the variant or breakpoint junction of 1000bp. We then assigned genotypes by calculating the fraction of reads mapped to the original reference sequence and the alternative contig. For indels ≤5bp, we used the GATK HaplotypeCaller to genotype the MiSeq data. The subsections below describe each of the validation experiments that were performed for different types and size classes of variations.

3.2.1 Indels of 1-20bp

Besides the validation assays as described previously for the GoNL release 1 dataset [2], we performed validation for an additional 96 indels, representative for the latest GoNL indel call set. Validation assays were performed on a single individual from the GoNL sample set. A successful PCR result was obtained for 86 out of 96 tested indels. For 84 out of 86 indels we observed reads corresponding to the expected indel allele.

3.2.2 21-100bp deletions

We have previously described validation for 96 deletions of 21-100bp [2]. We extended and reanalyzed these data and performed verification PCR assays and MiSeq sequencing for an additional 48 midsize deletions. Altogether, a total of 142 out of 144 21-100bp indels were confirmed.

3.2.3 Validation of 100bp+ deletions

We have previously described validation for 48 deletions larger than 100bp and observed a confirmatory rate of 46/48 [2]. Here, we performed validation assays for an additional 51 100+ deletions by PCR and MiSeq on a single individual from the GoNL sample set. Two assays failed and two deletions could not be confirmed.

3.2.4 Validation of tandem duplications

A total of 48 tandem duplications were tested using PCR and MiSeq on a single individual. We could confirm 41 out of 48 variants and failed to obtain a PCR product for 7 of them, resulting in a confirmation rate of 85.4%.

3.2.5 Validation of inversions

We identified a total of 76 inversions in the GoNL dataset. These were all subjected to experimental verification using PCR and MiSeq on diverse set of GoNL samples. Out of 76 tested inversion candidates, we confirmed 49, while the PCR assay failed for 27 variants, resulting in a confirmation rate of 64.5%.

3.2.6 Validation of mobile element insertions

We tested a 96 mobile element insertion (all Mobster calls) using PCR of one of the two breakpoint junctions. The assays were performed on a single individual of the GoNL dataset. We successfully validated 92 mobile element insertions, while 4 assays failed, resulting in a confirmation rate of 95.8%.

3.2.7 Validation of interchromosomal rearrangements

All 42 interchromosomal rearrangements detected in the GoNL cohort were independently tested by PCR and MiSeq. Based on 42 breakpoint PCR assays, we could confirm 35 interchromosomal changes, resulting in a confirmation rate of 83.3%. Recent data showed that interchromosomal rearrangements may occur as a result of gene retrotransposition insertion polymorphisms (GRIPs) [16]. We confirmed at least 10 GRIP events in our set of interchromosomal rearrangements (**Supplementary Data 2**).

3.2.8 Validation of large replacements

Using SOAPdenovo [13] we identified a set of 281 replacement SVs, which involve replacement of a genomic segment by an entirely different sequence (exceeding 150bp) (**Supplementary Data 3**). We tested the breakpoints for 96 of the replacement SVs by PCR and MiSeq. This successfully confirmed 94 variants, while 2 assays failed, resulting in a confirmation rate of 97.9%.

3.2.9 Validation of novel genomic segments

We tested 96 novel genomic segments, which were not present in the GRCh38 reference build. A total of 86 variants could be confirmed based on DNA from a single GoNL sample, while 10 assays did not result in a PCR product, resulting in a confirmation rate of 89.6%.

3.2.10 Validation of complex indels

We tested 10 complex indels and could confirm 8 of these, resulting in a confirmation rate of 80%.

3.3 Variant annotation

3.3.1 Functional annotation of Variants

The consensus events were annotated using a custom annotation pipeline as well as annovar [17], Great [18] and VEP [19]. To determine the genic content of the variants, an affected precedence annotation using RefGene was used (downloaded from UCSC on October 26th 2015). Furthermore variants were annotated (and in the case of balanced variants, their breakpoints) for overlap with DGV, dbVAR, dbSNP, OMIM disease genes, KEGG pathways, GO, MGI mouse phenotypes, repetitive elements segmental duplications, LINE, SINE, simple repeats, selfChain via UCSC as well as DNASE hypersensitivity sites.

3.3.2 Transmission Disequilibrium

A Transmission Disequilibrium Test (TDT) was performed on all autosomal deletions events across trios, which were Mendelian consistent. For each deletion event, transmitted reference and alternative alleles in the children were summed when at least one of the parents was heterozygous. Deletions with at least 20 transmitted alleles were binomially tested (two-sided) under the null hypothesis that the number of transmitted reference and alternative alleles was equal. P-values were subsequently adjusted via Benjamini & Hochberg.

3.3.3 Effects of SVs on gene expression

Whole blood paired end RNA-seq data is currently being generated for the Genome of The Netherlands samples. We used RNA-seq datasets for 115 samples that where available at the time of the analysis. Exon expression quantification was performed by HTSeq-count [20]. The exon definitions used for quantification were based on Ensembl (http://www.ensembl.org), version 71 with the extension that exons with overlapping parts were treated as single joint exons. Data were normalized using quantile normalization method. Variation of expression levels within each SV genotype group was checked for

normality. For each exon we tested whether the expression levels are correlated to the structural variant using the Spearman rank correlation test. We tested all inversions and mobile element insertions that overlapped exons or promoter region (up to 500 bp upstream of the transcription start site).

To explore whether new segments (described in section 3.1.13) are represented by expressed sequences, we BLASTed them to UniGene sequences (downloaded Sept 22, 2015). NCBI BLAST alignments with >99% identity were considered as representing genomic segments containing the corresponding UniGene entries.

3.3.4 Variant Hotspots

To identify genomic regions where deletions frequently occur, 1 Mb genomic bins across the primary sequence were created and the number of unique deletion events per bin counted based on the consensus set. Regions with significantly more events were identified based on a z-score greater than 2 and compared to those previously reported [4]. Likewise to identify hotspots across variant types, the analysis was repeated to include deletion, duplication, mobile element insertions and inversions. A cross variant hotspot was defined as a genomic region with a z-score greater than 2 across more than 3 variant classes.

Candidate hotspots were subsequently filtered based on accessibility for sequencing as previously reported [1].

3.3.5 Variant Novelty

To determine which SVs were known previously, we compared our call set to dbSNP, DGV, 1000 Genomes Data, CHM1 calls, and our previous public GoNL release [2,21–24]. Details are given below. In addition to the datasets named above, the novelty of the MEI call set was also compared to three additional studies [15,25,26].

3.3.5.1 Comparison to dbSNP

We compared simple and complex indel calls to dbSNP release 146 (released on 2015-11-04) by searching for exact matches of reference and alternative alleles between our and dbSNP polymorphisms.

3.3.5.2 Comparison to DGV

We compared our call set to release 2015-07-23 of the database of genomic variants (DGV, see footnote[1]) [24]. For deletions, we considered all DGV entries where "varianttype" was equal to "CNV" and "variantsubtype" was "deletion" or "loss". For duplications, we considered DGV entries with type "CNV" and subtype "duplication", "gain", or "tandem duplication". For MEIs, we used DGV entries with type "CNV" and subtype "mobile element insertion". For inversions, entries with type "OTHER" and subtype "inversion" were used. For inversions, duplications, and deletions longer than 100bp, we counted an event as a match if its center was at most 2000bp away from the center of the respective DGV event, and the length difference was at most 500bp. For the (more frequent) deletions 21-100bp, we used more stringent criteria to avoid false positives and counted matches with a center distance of at most 100bp and a length difference of at most 20bp. For MEIs (where a reliable length estimate is often not available), we counted DGV events as a match if the insertion point was at a distance of at most 500bp.

3.3.5.3 Comparison to 1000 Genomes Data

We compared our call set to the integrated SV release of phase 3 of the 1000 Genomes Project, see footnote[2]. We compared all deletions >20bp to 1000G events where SVTYPE matched DEL, CNV, DEL_ALU, DEL_HERV, DEL_LINE1, or DEL_SVA. Duplications were compared to SVTYPES matching DUP or CNV. Inversions were compared to records with SVTYPE=INV and MEIs were compared to SVTYPES matching ALU, LINE1, or SVA. As

[1] obtained from http://dgv.tcag.ca/dgv/docs/GRCh37_hg19_variants_2015-07-23.txt on 08/18/2015.
[2] obtained from ftp://ftp-trace.ncbi.nih.gov/1000genomes/ftp/phase3/integrated_sv_map/ALL.wgs.integrated_sv_map_v2.20130502.svs.genotypes.vcf.gz on 12/12/2015.

described above for DGV, matching was done based on center distance and length difference, using the same cutoffs.

3.3.5.4 Comparison to previous GoNL release

Previously [2], we have reported on a set of SNPs, simple indels, and deletions derived from the same NGS data. This call set is publicly available[3] as “release 5”. We also compared the call set reported here to this previous one and only report variants as novel that are not included in the previous set. For indels we required an exact match of coordinates and called reference and alternative alleles. For matching deletions >20bp, we used the same criteria as used above for DGV and 1000 Genomes.

3.3.5.5 Comparison to CHM1

We obtained calls from CHM1 [22] provided as three sets of events (insertions, deletions, and inversions) and compared them to our call set. For deletions and inversions, we used the same matching criteria as above. For insertions, the CHM1 data provides intervals (in a bed file). We counted a MEI as matching an insertion from CHM1 when the distance of our estimated insertion point was included in or at most 100bp away from one of the provided intervals.

3.4 GWAS correlation

3.4.1 Identification of significant (non)-GWAS SNP-deletion pairs

Autosomal SNP positions used by the HumanOmni2.5-8 v1.1 BeadChip are considered (2,332,914 in total). We downloaded all SNPs from the “Catalogue of Genome-Wide Association Studies” at https://www.genome.gov/26525384, on 08-06-2013. We removed SNPs whose description specified a non-Caucasian cohort and SNPs where the risk allele was not specified. Removing duplicates yielded 7,457 SNPs, all of which were shown to be

[3] at http://www.nlgenome.nl/?page_id=9.

associated with disease in Caucasian individuals, which in the following we will refer to as GWAS SNPs. In the following, we only consider data of the parents to ensure that the sample is (approximately) independent. Both the SNPs and the deletions were phased using MVNCall [27]. SNP and deletion variants with too low minor allele frequency (< 4%) were discarded, following the workflow described in Maurano *et al.*[18]. This filtering step reduced the number of autosomal GWAS, non-GWAS SNPs and deletions to 3,198, 1,259,007 and 21,685, respectively.

First, we tested for linkage disequilibrium (non-random association between alleles) in every GWAS SNP and deletion that are 1) on the same autosome, and 2) less than 1 million base pairs removed from each other (one CentiMorgan on average [Lodish et al., "Molecular Cell Biology"]), that is, associations are likely to be missed only in 1% of cases. The total number of GWAS SNP-deletion pairs that satisfied these constraints were 55,250. In order to assess association between each pair, we computed Pearson's coefficient of determination (R2) and applied Fisher's exact test (two-sided). We accounted for multiple testing by employing Benjamini-Hochberg's false discovery rate (FDR) control procedure with a q-value (expected FDR) set to 5% [28]. Of the initial GWAS SNP-deletion pairs considered, 14,033 (25.40%) were deemed statistically significant. **Supplementary Data 6** contains an annotated list of 115 GWAS SNP-deletion pairs that are deemed both materially/scientifically (operationalized as $R^2 \geq 0.8$) and statistically significant.

Secondly, we wanted to assess whether the relatively high percentage of significantly associated GWAS SNP-deletion pairs is potentially related to the GWAS status of the SNPs, or whether we would find comparable figures for non-GWAS SNPs (SNPs that, until now, have not been associated with disease). In order to account for potential confounding variables known (or theorized) to influence association strength (e.g., distance to the nearest transcription start site (TSS), the distance between variants), we employed the following sampling strategy inspired by the work in Maurano et al [29]: for every GWAS SNP-deletion pair, we randomly selected a similar non-GWAS SNP and deletion. Pairs are considered similar when 1) SNPs and deletion lie on the same autosome, 2) the SNPs are of the same

type (i.e., intergenic, intronic or exonic), 3) the difference in allele frequencies do not differ by more than 5%, 4) the SNPs are at a similar distance to the nearest TSS, i.e., the difference is less than 103 base pairs, and, finally, 5) the distances between SNP and deletion are similar (for which we used the same criterion as for the TSS distance). In case there is no non-GWAS SNP-deletion pair that satisfies these constraints, we randomly selected a non-GWAS SNP and deletion from the same autosome. This situation does not occur often, i.e., only 1.33% of the GWAS SNP-deletion pairs could not be matched. We performed exactly the same procedure on the set of non-GWAS SNP-deletion pairs as for the GWAS case: Fisher's exact test was applied to every pair and we controlled for false discoveries by applying Benjamini-Hochberg's procedure. This sample procedure was repeated 103 times, where for each run the percentage of non-GWAS SNP-deletion pairs that were deemed statistically significant was recorded. The result is shown in **Supplementary Figure 6**; the distribution is the result found for the matched non-GWAS SNP-deletion pairs (with a mean and standard deviation of, respectively, 19.14% and 0.20%). The dashed line represents the percentage of significant GWAS SNP-deletion pairs (25.40%). Note that none of the 103 samples approaches the percentage found for the GWAS SNPs, suggesting that, indeed, in the light of the confounding variables considered, deletions are significantly more frequent in LD with GWAS SNPs than non-GWAS SNPs.

The thresholds for the allele frequency (5%) and the distance between SNP and deletion and distance to the nearest TSS (both 104 base pairs) for classifying a non-GWAS deletion-pair as being similar to a GWAS SNP-deletion pair were picked rather arbitrarily. To investigate to what extent varying these values would influence our conclusion, we performed the same test with an allele frequency threshold of 2.5% and 10% and the distance thresholds set to 103 and $2.5x10^4$ base pairs. The results are shown in **Supplementary Figure 7**. Note that the observed percentage of significant GWAS SNP-deletion pairs continues to be much higher than for the matched non-GWAS SNP-deletion samples.

3.4.2 Tagging GoNL deletions

Autosomal SNP positions used by the HumanOmni2.5-8 v1.1 BeadChip are considered (2,332,914 in total). Deletions and SNPs with a minor allele frequency below the 4% were discarded, leaving 21,685 deletions and 1,262,205 SNPs. For every deletion we assessed the association strength with every SNP in the 1 million base pair vicinity by computing Pearson's coefficient of determination (R^2) and applying Fisher's (two-sided) exact test on the basis of the data of the parents. The SNP with the highest R^2 (of at least .8) and a *p*-value lower than 5% is regarded to be the most appropriate tag SNP for that particular deletion. The results can be found in **Supplementary Data 7**.

The code used for the identification of GWAS-SNP deletion correlation is available on Github: https://github.com/louisdijkstra/gonl-sv.

3.5 Genotyping indels and SVs

SV genotyping as performed for all SVs in the consensus call set described in Section 3.3. In the following we describe the performed steps in detail for each SV type.

3.5.1 A maximum likelihood model for genotyping insertions and deletions

The following model has been adopted from [Marschall & Schönhuth, to appear], where it was described for midsize deletions. Here, we will make use of the model for deletions of arbitrary length, and also for insertions that can be detected via paired-end read and split-read data signals. The model described in the following has been implemented as part of the CLEVER Toolkit (that also contains MATE-CLEVER).

Let G_0, G_1, G_2indicate the genotypes of an indel: G_0 denotes absence of the indel, G_1 denotes a heterozygous indel and G_2 a homozygous indel. In the following, we fix the region of interest, that is, the region that harbors the putative indel. Let A be a read alignment. Let R be a single read and **R** be the set of all reads that have an alignment with the region under

consideration. For R in **R**, let A(R) be the alignment of R with the region we would like to genotype.

Let **S** be a subset of reads from **R**. In slight abuse of notation, we formally consider **S** as the event that precisely the alignments of reads from **S** in the region of interest are correct, while all others do not align in the region. Hence,

$$P(\boldsymbol{S}) = \prod_{R \in \boldsymbol{S}} P\left(\{A(R)\ is\ correct\}\right) \cdot \prod_{R \notin \boldsymbol{S}} \left(1 - P(\{A(R)\ is\ correct\})\right)$$

is the corresponding probability. In the following, we consider a maximum likelihood (ML) setting, which in particular reflects that our prior belief in a genotype to be correct is the same for all types:

$$P(G_0) = P(G_1) = P(G_2) = 1/3$$

This assumption is necessary for an efficient computation scheme, because a more flexible Bayesian approach would only yield a scheme that is exponential in |**R**|, the number of reads that align to the region of interest. If |**R**| exceeds 20, this becomes infeasible.

We are interested in maximizing (j=0,1,2)

$$P\left(G_j|\boldsymbol{R}\right) \propto P\left(G_j, \boldsymbol{R}\right) = \sum_{\boldsymbol{S} \subset \boldsymbol{R}} P\left(\boldsymbol{S}\right) \cdot P\left(G_j|\boldsymbol{S}\right)$$

(1)

By considering probabilities P(**S**), we take alignment uncertainty due to the usual reasons like multiply mapped reads and alignment artifacts, which occur particularly often in indel-affected regions, into account. Let K:=|**R**| be the number of reads that align to the region to be genotyped. By Bayes' formula, equation (1) further implies that

$$P(G_j|\boldsymbol{S}) \propto P(\boldsymbol{S}|G_j) = \prod_{R\in\boldsymbol{S}} P\left(\{A(R)\ is\ correct\}|G_j\right) \cdot \prod_{R\notin\boldsymbol{S}} P\left(G_j|\{A(R)\ is\ not\ correct\}\right)$$

$$\ldots \propto \prod_{R\in\boldsymbol{S}} P\left(G_j|\{A(R)\ is\ correct\}\right) \cdot \prod_{R\notin\boldsymbol{S}} P\left(G_j|\{A\ is\ not\ correct\}\right)$$

(2)

Note that we also assume that reads were generated independently of each other, which is a reasonable assumption. From equation (2), we conclude that

$$P(G_j|\boldsymbol{R}) \propto \sum_{\boldsymbol{S}\subset\boldsymbol{R}} P\,(\boldsymbol{S}) \cdot \prod_{R\in\boldsymbol{S}} P\left(G_j|\{A(R)\ correct\}\right) \cdot \prod_{R\notin\boldsymbol{S}} P\left(G_j|\{A(R)\ not\ correct\}\right)$$

$$\ldots = \sum_{\boldsymbol{S}\subset\boldsymbol{R}} \prod_{R\in\boldsymbol{S}} P\,(\{A(R)\ correct\})P(G_j|\{A(R)\ correct\}) \cdot\ldots$$

$$\ldots \cdot \prod_{R\notin\boldsymbol{S}} (1 - P(\{A(R)\ correct\}))\, P(G_j|\{A\ not\ correct\})$$

$$\ldots = \prod_{R\in\boldsymbol{R}} [P(\{A(R)c\ orrect\})P(G_j|\{A(R)\ correct\}) + \ldots$$

$$\ldots + (1 - P(\{A(R)c\ orrect\}))P(G_j|\{A(R)\ not\ correct\})]$$

where the second row results from expanding the third row. The last term, finally, can be computed in time linear in the number of reads **R**, which had been our goal. We use this formula for genotyping insertions and deletions of arbitrary length, that is, including insertion and deletion SVs.

It remains to compute reasonable probabilities $P(G_j|\{A\ correct\})$and $P(G_j|\{A\ not\ correct\})$ for read alignments A. While $P(G_j|\{A\ not\ correct\}) = P(G_j)$ because the read that underlies A does not stem from the region, hence has no influence on the respective genotype, computation of terms $P(G_j|\{A\ correct\})$require further reasoning. One has to distinguish between two types of evidence for an indel given that A is correct:

- *Split-read evidence*: A aligns with the region to be genotyped such that one read end stretches across the breakpoint(s) of the putative indel
- *Internal-segment based evidence*: A aligns with the region of interest such that the internal segment of the read stretches across the breakpoint(s) of the putative indel

3.5.1.1 Split-read evidence

Let us first consider the case of no alignment uncertainty, that is, all alignments are correct and indicate indel breakpoints accurately. We focus on deletions in the following, while everything applies analogously, mutatis mutandis, for insertions. Let D be the deletion to be genotyped and let A be an alignment where, for example, A_L, the alignment of the left end of the read stretches across the breakpoints of D. Under the assumption of no alignment uncertainty, that is, A is correct with probability one, we obtain that A stems from a chromosomal copy that is affected by D if and only if A has a gap such that the left breakpoint of the gap, w_A, and the right breakpoint of the gap, z_A precisely agree with the left and right breakpoint of D. If the gap in A_L disagrees with the deletion breakpoints or there is no gap, the read of A stems from a chromosomal copy that is not affected by the deletion with probability one.

Let A be an alignment with a split/gap that agrees with D. By the above considerations, the read of A stems from a chromosomal copy that is affected by D. By Bayes' formula, and the formulas from above,

$$P\big(G_j|\{A\ correct\}\big) \propto P\big(\{A\ correct\}|G_j\big)$$

First, $P(\{A\ correct\}|G_0) = 0$, because if A has a gap, it cannot stem from a region without deletion, as is expressed by G_0, and $P(\{A\ correct\}|G_1) = 1/2 \cdot P(\{A\ correct\}|G_0) + P(\{A\ correct\}|G_2)$, which reflects that one first randomly selects one of the two chromosomal copies, only one of which is affected by D, and

then generates the read from it. The case of B being an alignment in disagreement with D is treated analogously, where in this case $P(\{B\ correct\}|G_2) = 0$. Transforming this into a posterior probability then yields

$$P(G_2|\{A\ correct\}) = 2/3, P(G_1|\{A\ correct\}) = 1/3, P(G_0|\{A\ correct\}) = 0$$

and

$$P(G_2|\{B\ correct\}) = 0, P(G_1|\{B\ correct\}) = 1/3, P(G_0|\{B\ correct\}) = 2/3$$

In general, the assumption of no alignment uncertainty does not apply. In fact, split-read (gapped) alignments can be affected by several sources of errors, the most evident of which are repetitive areas, such that both position and length of alignment splits (=gaps) disagree with the exact positions and the length of the true variants. However, despite these (minor) disagreements in terms of split location and length, the split corresponds to the variant. Therefore, we set $C(A) := (w_A + z_A)/2$ to be the centerpoint and $L(A) := z_A - w_A$ to be the length of the gap in A (for insertions, C(A) is the only breakpoint). We say that A supports D, which is specified by its centerpoint C(D) (= the mean of its breakpoints) and its length L(D), if and only if

$$|C(D) - C(A)| \leq 50 \quad \text{and} \quad |L(D) - L(A)| \leq 20.$$

While these values may seem large, they are well supported by considerations on the uncertainty of (split-)alignments [9,30].

3.5.1.2 Internal Segment-Based Evidence

Internal-segment-based evidence is provided by evaluating the empirical distribution on fragment length, which depends on the library from which the read stems. Here, we consider this distribution approximately Gaussian, which is a reasonable assumption for the vast majority of GoNL individuals. In general, all of the following considerations can be easily generalized to arbitrary, also non-Gaussian empirical distributions. Let D be the deletion to be genotyped and let C(D) be its centerpoint. Let R be the read of A where $x_A < C(D) < y_A$, that is, the alignment interval of A, which is the gap between A_L, the alignment of the left end, and A_R, the alignment of the right end, contains the counterpoint of the deletion D. Note that choosing centerpoints as anchors for the following considerations has advantages, as was explained in [9,30]. Let μ and σ be mean and standard deviation of the internal segment size distribution of the library R is from, which assumed to be Gaussian. So, internal segment length, as a random variable X, is distributed as the Gaussian distribution

$$X \sim N_{\mu,\sigma}.$$

There are two cases: first, alignments A whose reads stem from a chromosomal copy that is not affected by D, and second, alignments B whose reads stem from a chromosomal copy that is affected by D. We obtain

$I(A) \sim N_{\mu,\sigma}$ and $I(B) \sim N_{\mu+L,\sigma}$,

where the second case reflects that the alignment interval contains the deletion of length L.

We compute

$P(\{Acorrect\} \vee G_0) \propto N_{\mu,\sigma}\big(I(A)\big)$ and $P(\{Acorrect\}|G_2) \propto N_{\mu+L,\sigma}\big(I(A)\big)$

as appropriate densities for the cases of no variant and a homozygous variant.

Let

$$Z := 3/2\left(N_{\mu,\sigma}\big(I(A)\big) + N_{\mu+L,\sigma}\big(I(A)\big)\right)$$

a normalization factor.

In analogy to considerations for the split-read case, we arrive at

$P(G_2|\{A\ correct\}) = 2/3, P(G_1|\{A\ correct\}) = 1/3, P(G_0|\{A\ correct\}) = 0$

and

$P(G_2|\{B\ correct\}) = 0, P(G_1|\{B\ correct\}) = 1/3, P(G_0|\{B\ correct\}) = 2/3$

as an appropriate probability distribution for reads whose alignments span the breakpoints of deletions by their internal segments.

The procedure described above is implemented as part of MATE-CLEVER [9], which can also use prior information in form of the Mendelian laws, if the input consists of multiple, ancestry-related genomes. In order to genotype, MATE-CLEVER collects all (split-read and regular) alignments in a region, and uses them for carrying out the computations described above.

3.5.2 Mid-size deletions (20-10000bp)

For the consensus deletion calls in this length range, the respective regions (deletions +/-750bp) were extracted from the BAM files for all samples, converted to FASTQ format, and

aligned using LASER [9], version v2.0-rc1-20-gfea4980, with the following command line: “laser -M 10000 --extra-sensitive -T 8 -w 0.1 --keep_raw_bam --dont-recalibrate -S fastq/${family}.${ind}.split.fastq.gz ref.fasta 1.fastq.gz 2.fastq.gz output-prefix”.

To refine breakpoint coordinates, we collected all putative deletions found by LASER from all samples (i.e. all deletions in any read in any sample). To compute a weight for every such deletion candidate, we computed the sum of mapping probabilities (derived from MAPQs) for all supporting reads across all samples. This weight can be interpreted as the expected support. For each deletion in the consensus set, we then picked the LASER deletion with the largest weight within a center distance of 10bp and a length difference of 5bp. For IMPRECISE deletions in the consensus set (i.e. those predicted by a method not having single-basepair resolution), we allowed for a center distance of up to 100bp and a length difference of up to 50bp. In all following steps, we used the refined breakpoint coordinates obtained in this way.

For each family, we compute a list of putative SNPs with an expected support (computed as in the previous step) of 3 or more. This set of putative SNPs and the breakpoint-refined consensus set of deletions where used to recalibrate each LASER BAM file (one for each sample) using the following command line: “laser-recalibrate -csMRr -H org.bam -V deletions.list -S snps.list family.insert-sizes”, where family.insert-sizes is a file containing the estimated insert size mean and standard deviation for all libraries / read groups present in the respective family.

For each family, we ran the genotyper (also part of the CLEVER Toolkit, version v2.0-rc3-47-gaa50e36) [10] as follows: “genotyper --dont-prioritize --min_phys_cov 5 --min_gq 10 --gq-present 20 --denovo_threshold 0 --variant_prior 0.49 --mapq 30” , where deletions.sorted.list refers to the breakpoint-refined deletions obtained above, sorted by expected support on the full cohort. The file samples.list contains information on the samples part of the respective family, on the location of the needed recalibrated LASER BAM files and on the family relationships to be used during genotyping.

The resulting VCFs for each family are merged using vcf-merge, which is part of vcftools [31],

to create a VCF for the full panel.

Phasing requires well-calibrated genotype likelihoods. We therefore conducted a simulation study to recalibrate GLs. We created three kinds of simulated diploid genomes, one that does not contain any midsize deletions (i.e. homozygous for the reference allele at all midsize deletion loci), one that is heterozygous for all midsize deletions (each one independently put at random onto one of the two haplotypes), and one that is homozygous for all midsize deletion alleles. We additionally added SNPs and indels to these three simulated genomes by randomly drawing SNP and indel alleles from the GoNL panel according to their AFs. We repeated this procedure 10 times to create a total of 30 simulated diploid genomes. For all these genomes, we simulated Illumina reads using the simseq program [32], matching the coverage found in the GoNL. The resulting reads were aligned to the reference genome using LASER with the same parameters as described above. Subsequent genotyping using the CLEVER Toolkit was also performed as described above. For each deletion, we created a confusion matrix, counting the number of times we observed genotyped G_{pred} (predicted by our genotype caller) when the true genotype was G_{true}, for all nine possible combinations of genotypes. Based on these counts, we estimated the empirical conditional probabilities $P(G_{true}|G_{pred})$, based on the assumption that $P(G_{true}|G_{pred})$ is proportional to $P(G_{pred}|G_{true})$. In the absence of observations (i.e. when the number of observed genotypes G_{pred} is zero), we assume uniform conditional probabilities (corresponding to a flat prior). Using these deletion specific tables of conditional probabilities, we recalibrated all genotype likelihoods by computing posterior likelihoods.

3.5.3 Long deletions (10kbp and longer)

In principle, the genotyping of long deletions proceeded like the genotyping of mid-size deletions described above, with the following slight adaptations:

- The analysis was done at a later point in time using the (then current) version v2.0-rc3-53 g7b13d29 of the CLEVER Toolkit. No major changes happened compared to the versions used for mid-size deletions, just slight fixes/usability improvements.

- Due to the size of the events, we did not extract the full-length deletions from the original BAM files, but used a +/- 1000bp window around start position, center position, and end position of each deletion, which is sufficient for genotyping.
- LASER was run with the following parameters: "-T 7 --dont-recalibrate -w 0.1 --keep_raw_bam --core-options "--soft_clip -A 12 --anchor_distance 1000 --max_anchors 500 --max_anchor_pairs 5000 --max_span 1500000 --max_insert 1500500"".

3.5.4 Inversions

To genotype the inversions in our consensus set, we used DELLY [33], version 0.5.9. Since precise breakpoint coordinates were not available for all consensus inversions, we did not run delly in "genotyping mode", but used the "discovery mode" as follows:

1. We extracted reads from a region of +/-1000bp around each inversion from all samples and mapped them with BWA MEM [34], version 0.7.10-r789, to create one BAM file per sample.
2. We ran DELLY on these files in parallel with the following command line: "delly -t INV -g ref.fasta -o output.vcf sample1.bam … sample769.bam"
3. For each inversion in our consensus set, we consider all inversions reported by DELLY for which both breakpoint coordinates are within a +/-500bp window around the original coordinates. Out of those candidates, we retain the best one for each consensus inversion. The best one is selected by choosing according to the following criteria, in the listed order: we prefer calls with a FILTER value of "PASS" over those with a "LowQual", we prefer PRECISE over IMPRECISE, we count the number of reported genotypes consistent with our previous present/absent annotations and prefer calls with higher consistency, and we finally prefer calls with a lower distance to the original consensus inversion. Inversions with an AN value of 200 or larger, corresponding to successful genotyping in at least 100 samples, where marked as successfully genotyped. This applied to 69 out of all 84 inversions in the consensus

set.

3.5.5 Translocations

Genotyping of translocation was done analogously to genotyping inversions (see previous section).

3.5.6 Duplications

Genotyping of duplications proceeded mostly like for inversions as described above, with the following differences:

1. Due to the large number of duplications in our consensus set (1738), we split the BAM files by duplication candidate and ran DELLY on each region separately, using the command line "delly -t DUP -g ref.fasta -o output.vcf sample1.bam … sample769.bam". We then concatenated the resulting VCF files to create one file containing all candidates produced by DELLY.
2. We matched consensus duplications to DELLY prediction, again using a maximum breakpoint distance of 500 and resolved ambiguities as follows: we prefer calls with a FILTER value of "PASS" over those with a "LowQual", we prefer PRECISE over IMPRECISE, we prefer calls with a lower distance to the original consensus duplication, and finally we prefer calls with a lower length difference.

3.5.7 Mobile Element Insertions

Genotyping of mobile element insertions (MEIs) was done via a logistic regression model, using concordant pair percentage and the presence of a Mobster call as predictor. For each MEI a search window of 400bp surrounding the insertion was used to identify concordant and discordant pairs to calculate the concordant pair percentage. Pairs were considered concordant when i) the pair was flagged as properly paired by the aligner ii) none of the ends were clipped by more than 10bp and iii) the fragment spanned the insertion coordinate. Pairs were considered discordant when i) they were marked as improperly paired by the aligner

and ii) one end was aligned left of the insertion and was aligned on the positive strand or one end was aligned right of the insertion and on the negative strand. Positive aligned ends were allowed to have an alignment end up to 20bp right of the insertion and negative aligned ends were allowed to have an alignment start up to 20bp left of the insertion to allow for TSDs. The model was subsequently trained on an independent set of 360 PCR validated MEIs from NA12878 (n = 185 homozygous reference, n = 134 heterozygous insertions, n = 41 homozygous insertions) [15] using WEKA 3 [35]. Concordant percentages for this sample were calculated by downsampling the high coverage WGS bam from NA12878 [36] to 15% of its original coverage, matching the median coverage of the GoNL cohort. In addition Mobster was run on the downsampled sample, requiring at least 2 supporting reads. The trained model was then applied to 748 GoNL samples, genotype probabilities were rescaled by adding $1x10^{-5}$ to all probabilities and log10 transformed.

3.6 Creating an SV-integrated reference panel

3.6.1 Phasing SVs using MVNCall

We used the same haplotype scaffolds as previously described [2] to phase SVs onto the already phased sets of SNPs and indels. The scaffold contains sites present on Omni2.5M chips. Refer to the supplement of Francioli *et al* [2], Section 12, for details on how it was created. For phasing, we used MVNcall [27] version 1.1. Apart from PED files (containing the family/trio structure) and scaffolds, we used the genotype likelihoods (GLs) reported by the CLEVER Toolkit (for deletions) [10], by DELLY (for inversions, duplications, and translocations) [33], by Mobster [14] (for mobile element insertions), and by GATK's HaplotypeCaller (for complex indels) as input. Before phasing, the GLs were regularized so as to avoid too low probabilities as follows. We added a value of 10^5 to each of the three probabilities for genotypes (RR, AR, and AA) and renormalized the sum to one. The so transformed GLs are passed to MVNcall [27] for phasing using the following command line: "mvncall --sample-file

samples.list --ped-file samples.ped --k 100 --iterations 50 --add-gls --int 0 300000000 --scaffold-file scaffolds.haps --glfs input.vcf --o output.vcf". We ran MVNcall in this way for each chromosome and for each SV type separately.

3.6.2 Imputing SVs in independent Dutch individuals using IMPUTE2

In order to assess the quality of our reference panel with respect to its structural variants (see [37] for an encompassing study on the quality of the panel in terms of SNPs), we ran IMPUTE2 [38] on two independent Dutch individuals and analyzed the resulting genotypes. We aimed at a scenario where one could compare the imputed genotypes with true genotypes. This differs from the evaluation in Deelen et al 2014 [37] who focused on classical imputation metrics [39] as a basis for evaluation, which requires sufficiently many individuals, but does not use true genotypes. Since the application of imputation metrics for SVs has not been discussed before in the literature, we opted for a scenario where one could juxtapose imputed genotypes with (highly likely) true genotypes.

3.6.2.1 Generating ground truth structural variant genotypes.

For genotyping structural variants in the two individuals, we analyzed the available whole-genome sequencing data of the individuals, which were Illumina HiSeq paired-end reads at 17x and 19.5x coverage respectively, aligned against reference genome GRCh37 using Bowtie2 v2.2.4 [40], followed by marking duplicated reads and indel realignment. For genotyping SVs, we used the same tools with the same setting as for the GoNL panel, see Section **3.5**; that is, we used MATE-CLEVER [9] for genotyping deletions, DELLY [33] for genotyping inversions, duplications and translocations, Mobster [14] for genotyping mobile element insertions, and GATK's HaplotypeCaller for genotyping complex indels. We ran all these tools in genotyping mode - and not in discovery mode - on the structural variants that belong to the panel. This yielded a set of read-based genotyped SVs that is in 1-to-1-correspondence with the SVs one can impute, in particular also in terms of breakpoint

annotations, which is important for an unbiased evaluation. We then further filtered the SVs for genotypes where the maximum genotype likelihood (GL) was 0.85 for mobile element insertions and 0.999 for all other SV types. We will henceforth refer to the resulting set of SVs and their genotypes as "gold standard genotypes".

3.6.2.2 Phasing and imputing structural variants in the two independent individuals

Workflow: We used IMPUTE2 [38], a widely-used state-of-the-art tool, for imputing the SVs based on SNP genotypes. The novelty in the following is that we will impute SVs and not only SNPs and indels.

Genotyping and phasing SNPs in the individuals. Genotyping SNPs was done using GATK's HaplotypeCaller on the SNPs present on an Affymetrix 6 SNP chip. These SNP genotypes are the basis for phasing and imputation in the following. We then applied SHAPEIT2 (v2.r790) [41] to the SNP genotypes of the two Dutch samples for phasing the SNP genotypes, which reflects best-practice recommendations for imputation (see the IMPUTE2 manual at https://mathgen.stats.ox.ac.uk/impute/impute_v2.html).
As the underlying reference panel for this step, we used the GoNL release 4 SNPs [2]. We recall that this SNP-only panel is consistent with the SV-integrated panel that we present in this paper. We ran the following command for each chromosome:

"shapeit -M genetic-map.txt --input-gen study-data.gen study-data.sample --input-ref ref-panel.hap ref-panel.legend ref-panel.sample -O output --no-mcmc --exclude-snp snp.strand.exclude",

where:

- genetic-map.txt is the 1000 Genomes genetic map, downloaded from https://mathgen.stats.ox.ac.uk/impute/1000GP_Phase3.html (23-04-2015),

- study-data.gen and study-data.sample contain the unphased SNP genotypes of the 2 study individuals in gen/sample format,
- ref-panel.hap, ref-panel.legend, and ref-panel.sample represent the GoNL release 4 SNP reference panel in hap/leg/sample format, and
- snp.strand.exclude is a file containing SNP positions to be excluded, obtained by running SHAPEIT2 [41] in check-mode first.

SHAPEIT2 produces phased haplotypes in haps/sample format, to be used as input for impute2.

Some SNP positions had to be excluded because they did not align well; a list of the 2013 such positions was obtained by running SHAPEIT2 in check-mode first. The resulting haplotypes contained 519277 SNP positions as a basis for further imputation.

Imputation. The different SV types (complex indels, deletions, duplications, MEIs, and inversions) were imputed separately and each chromosome was processed in chunks of 5Mb (using -int to specify the positions to include). We ran IMPUTE2 version 2.3.2 using the following command line:

"impute2 -m genetic-map.txt -h ref-panel.hap -l ref-panel.legend -use_prephased_g -known_haps_g phased-study-sample.haps -int <lower> <upper> -o output",

where:

- genetic-map.txt is the same genetic map as used for SHAPEIT2,
- ref-panel.hap and ref-panel.legend are the reference panels containing the desired SV haplotypes (for the desired SV type), merged with the SNP haplotypes,
- phased-study-sample.haps are the phased study haplotypes obtained from SHAPEIT2, and

- lower and upper are integers to indicate the genomic region to be studied (we use the full chromosome).

This resulted in imputation-based genotype likelihoods (GL's) for each SV. We then filtered these GL's for SV's that correspond to SV's where gold standard genotypes, as described above, were available.

Comparing genotypes / allele dosages. Let V be a placeholder for single SV's, and let **V** be all SV's considered (stratified by type or panel-based allele frequency, for example). For each V, we compare the imputed (SNP- and panel-based) genotype likelihood $q_{V,0}, q_{V,1}, q_{V,2}$ which correspond to the imputation-based probabilities that the V is not present (0), heterozygous (1) or homozygous (2), with the directly read-based, carefully filtered "gold standard genotype" likelihoods $p_{V,0}, p_{V,1}, p_{V,2}$ which reflect the ground truth probabilities that V is absent (0), heterozygous (1) or homozygous (2); note that one of the $p_{V,i}$ is at least 0.999 as per the filtering criteria (0.85 for MEI's). Let V be a placeholder for single SV's. We then compute the probability P_V that the imputed genotypes agree with the ground truth as

$$P_V = \sum_{i=0}^{2} p_{V,i}\, q_{V,i}$$

Averaging P_V over all SV's **V** under consideration (for example, **V** represents all duplications of allele frequency below 0.05, **V** can vary depending on the context), that is computing

$E_{conc}(V) = (\sum P_V)/|V|$

Similarly, $E_{disc}(V) = 1 - E_{conc}(V)$ is the expected rate of discordance of the imputed genotypes with the ground truth among the variants **V**. E_{conc} and E_{disc} are the quantities

referred to as discordance and concordance in the main text. In the following, c is a lower bound for the maximum imputed genotype likelihood, that is, for example, for $c = 0.99$, we filter the variants **V** considered for variants whose maximum imputed genotype (either $q_{V,0}, q_{V,1}$ or $q_{V,2}$) is at least 0.99.

As a sanity check, we also emulated the following naïve imputation algorithm. Here, values $q_{V,0}, q_{V,1}$ and $q_{V,2}$ reflect the empirical statistics on genotype counts in the reference panel. That is, if there are $n_{V,0}$ individuals among the 499 panel individuals who do not carry the variant V, $n_{V,1}$ individuals with a heterozygous variant V allele and $n_{V,2}$ with a homozygous variant V allele, then $q_{V,0} = n_{V,0}/499, q_{V,1} = n_{V,1}/499$ and $q_{V,2} = n_{V,2}/499$.

We juxtapose the corresponding concordance/discordance rates with the ones obtained from IMPUTE2-based imputation, as a minimum requirement for a real panel-based imputation of SVs. The results are shown in **Supplementary Figure 11**.

4 Supplementary References

1. Kloosterman, W. P. *et al.* Characteristics of de novo structural changes in the human genome. *Genome Res.* **25,** 792–801 (2015).
2. Francioli, L. C. *et al.* Whole-genome sequence variation, population structure and demographic history of the Dutch population. *Nat. Genet.* **46,** 818–25 (2014).
3. Ye, K., Schulz, M. H., Long, Q., Apweiler, R. & Ning, Z. Pindel: a pattern growth approach to detect break points of large deletions and medium sized insertions from paired-end short reads. *Bioinformatics* **25,** 2865–71 (2009).
4. Mills, R., Luttig, C. & Larkins, C. An initial map of insertion and deletion (INDEL) variation in the human genome. *Genome Res.* **16,** 1182–1190 (2006).
5. Kloosterman, W. P. *et al.* Chromothripsis as a mechanism driving complex de novo structural rearrangements in the germline. *Hum. Mol. Genet.* **20,** 1916–1924 (2011).
6. Chen, K. *et al.* BreakDancer: an algorithm for high-resolution mapping of genomic structural variation. *Nat. Methods* **6,** 677–681 (2009).
7. Abyzov, A., Urban, A. E., Snyder, M. & Gerstein, M. CNVnator: An approach to discover, genotype, and characterize typical and atypical CNVs from family and population genome sequencing. *Genome Res.* **21,** 974–984 (2011).
8. Coe, B. P., Chari, R., MacAulay, C. & Lam, W. L. FACADE: A fast and sensitive algorithm for the segmentation and calling of high resolution array CGH data. *Nucleic Acids Res.* **38,** (2010).

9. Marschall, T., Hajirasouliha, I. & Schönhuth, A. MATE-CLEVER: Mendelian-inheritance-aware discovery and genotyping of midsize and long indels. *Bioinformatics* **29,** 3143–3150 (2013).

10. Marschall, T. *et al.* CLEVER: Clique-enumerating variant finder. *Bioinformatics* **28,** 2875–2882 (2012).

11. Köster, J. & Rahmann, S. Snakemake-a scalable bioinformatics workflow engine. *Bioinformatics* **28,** 2520–2522 (2012).

12. Handsaker, R. E., Korn, J. M., Nemesh, J. & McCarroll, S. A. Discovery and genotyping of genome structural polymorphism by sequencing on a population scale. *Nat. Genet.* **43,** 269–276 (2011).

13. Luo, R. *et al.* SOAPdenovo2: an empirically improved memory-efficient short-read de novo assembler. *Gigascience* **1,** 18 (2012).

14. Thung, D. T. *et al.* Mobster: accurate detection of mobile element insertions in next generation sequencing data. *Genome Biol.* **15,** 488 (2014).

15. Stewart, C. *et al.* A comprehensive map of mobile element insertion polymorphisms in humans. *PLoS Genet.* **7,** (2011).

16. Ewing, A. D. *et al.* Retrotransposition of gene transcripts leads to structural variation in mammalian genomes. *Genome Biol.* **14,** R22 (2013).

17. Wang, K., Li, M. & Hakonarson, H. ANNOVAR: Functional annotation of genetic variants from high-throughput sequencing data. *Nucleic Acids Res.* **38,** (2010).

18. McLean, C. Y. *et al.* GREAT improves functional interpretation of cis-regulatory regions. *Nat. Biotechnol.* **28,** 495–501 (2010).

19. McLaren, W. *et al.* Deriving the consequences of genomic variants with the Ensembl API and SNP Effect Predictor. *Bioinformatics* **26,** 2069–2070 (2010).

20. Anders, S., Pyl, P. T. & Huber, W. HTSeq – A Python framework to work with high-throughput sequencing data HTSeq – A Python framework to work with high-throughput sequencing data. 0–5 (2014).

21. Sudmant, P. H. *et al.* An integrated map of structural variation in 2,504 human genomes. *Nature* **526,** 75–81 (2015).

22. Chaisson, M. J. P. *et al.* Resolving the complexity of the human genome using single-molecule sequencing. *Nature* **advance on,** (2014).

23. Sherry, S. T. *et al.* dbSNP: the NCBI database of genetic variation. *Nucleic Acids Res.* **29,** 308–311 (2001).

24. MacDonald, J. R., Ziman, R., Yuen, R. K. C., Feuk, L. & Scherer, S. W. The Database of Genomic Variants: a curated collection of structural variation in the human genome. *Nucleic Acids Res.* **42,** D986–92 (2014).

25. Hormozdiari, F. *et al.* Alu repeat discovery and characterization within human genomes. *Genome Res.* **21,** 840–9 (2011).

26. Lee, E. *et al.* Landscape of somatic retrotransposition in human cancers. *Science* **337,** 967–71 (2012).

27. Menelaou, A. & Marchini, J. Genotype calling and phasing using next-generation sequencing reads and a haplotype scaffold. *Bioinformatics* **29,** 84–91 (2013).

28. Benjamini, Y. & Hochberg, Y. Controlling the False Discovery Rate: A Practical and Powerful Approach to Multiple Testing. *J. R. Stat. Soc. Ser. B* **57,** 289 – 300 (1995).

29. Maurano, M. T. *et al.* Systematic Localization of Common Disease-Associated Variation in Regulatory DNA. *Science (80-.).* **337,** 1190–1195 (2012).

30. Wittler, R., Marschall, T., Schonhuth, A. & Makinen, V. Repeat- and Error-Aware

Comparison of Deletions. *Bioinformatics* 1–8 (2015).

31. Danecek, P. *et al.* The variant call format and VCFtools. *Bioinformatics* **27,** 2156–8 (2011).

32. Benidt, S. & Nettleton, D. SimSeq: A Nonparametric Approach to Simulation of RNA-Sequence Datasets. *Bioinformatics* **31,** 2131–2140 (2015).

33. Rausch, T. *et al.* DELLY: Structural variant discovery by integrated paired-end and split-read analysis. *Bioinformatics* **28,** (2012).

34. Li, H. & Durbin, R. Fast and accurate short read alignment with Burrows-Wheeler transform. *Bioinformatics* **25,** 1754–1760 (2009).

35. Hall, M. *et al.* The WEKA Data Mining Software : An Update. *SIGKDD Explor.* **11,** 10–18 (2009).

36. ftp://ftp-trace.ncbi.nih.gov/1000genomes/ftp/technical/working/20120117_ceu_trio_b37_decoy/CEUTrio.HiSeq.WEx.b37_decoy.NA12878.clean.dedup.recal.20120117.bam.

37. Deelen, P. *et al.* Improved imputation quality of low-frequency and rare variants in European samples using the 'Genome of The Netherlands'. *Eur. J. Hum. Genet.* 1–6 (2014).

38. Howie, B. N., Donnelly, P. & Marchini, J. A flexible and accurate genotype imputation method for the next generation of genome-wide association studies. *PLoS Genet.* **5,** (2009).

39. Marchini, J. & Howie, B. Genotype imputation for genome-wide association studies. *Nat. Rev. Genet.* **11,** 499–511 (2010).

40. Langmead, B. & Salzberg, S. L. Fast gapped-read alignment with Bowtie 2. *Nat. Methods* **9,** 357–359 (2012).

41. Delaneau, O., Howie, B., Cox, A. J., Zagury, J. F. & Marchini, J. Haplotype estimation using sequencing reads. *Am. J. Hum. Genet.* **93,** 687–696 (2013).
